# Sodium New Houttuyfonate Inhibits *Candida albicans* Biofilm Formation by Inhibiting the Ras1-cAMP-Efg1 Pathway Revealed by RNA-seq

**DOI:** 10.3389/fmicb.2020.02075

**Published:** 2020-08-25

**Authors:** Jiadi Wu, Daqiang Wu, Yeye Zhao, Yuanqing Si, Longfei Mei, Jing Shao, Tianming Wang, Guiming Yan, Changzhong Wang

**Affiliations:** ^1^Department of Pathogenic Biology and Immunology, College of Integrated Chinese and Western Medicine, Anhui University of Chinese Medicine, Hefei, China; ^2^Research Institute of Integrated Traditional Chinese and Western Medicine, Anhui University of Chinese Medicine, Hefei, China; ^3^Key Laboratory of Chinese Herbal Compound Formula in Anhui Province, Hefei, China

**Keywords:** *Candida albicans*, sodium new houttuyfonate, anti-adhesive, anti-biofilm, Ras1-cAMP-Efg1 pathway

## Abstract

Here, we aim to investigate the antifungal effect and mechanism of action of sodium new houttuyfonate (SNH) against *Candida albicans*. Microdilution analysis results showed that SNH possesses potent inhibitory activity against *C. albicans* SC5314, with a MIC_80_ of 256 μg/mL. Furthermore, we found that SNH can effectively inhibit the initial adhesion of *C. albicans*. Inverted microscopy, crystal violet staining, scanning electron microscopy and confocal laser scanning microscopy results showed that morphological changes during the transition from yeast to hypha and the biofilm formation of *C. albicans* are repressed by SNH treatment. We also found that SNH can effectively inhibit the biofilm formation of clinical *C. albicans* strains (Z103, Z3044, Z1402, and Z1407) and SNH in combination with fluconazole, berberine chloride, caspofungin and itraconazole antifungal agents can synergistically inhibit the biofilm formation of *C. albicans*. Eukaryotic transcriptome sequencing and qRT-PCR results showed that SNH treatment resulted in significantly down-regulated expression in several biofilm formation related genes in the Ras1-cAMP-Efg1 pathway (*ALS1, ALA1, ALS3, EAP1, RAS1, EFG1, HWP1*, and *TEC1*) and significantly up-regulated expression in yeast form-associated genes (*YWP1* and *RHD1*). We also found that SNH can effectively reduce the production of key messenger cAMP in the Ras1-cAMP-Efg1 pathway. Furthermore, using *Galleria mellonella* as an *in vivo* model we found that SNH can effectively treat *C. albican*s infection *in vivo*. Our presented results suggest that SNH exhibits potential antibiofilm effects related to inhibiting the Ras1-cAMP-Efg1 pathway in the biofilm formation of *C. albicans*.

## Introduction

Fungal infections caused by *Candida albicans*, an opportunistic fungal pathogen (Naglik et al., 2006), are a growing threat in the immune deficient population (Wang et al., 2018), and *C. albicans* is the species most frequently implicated in different forms of invasive candidiasis (Rodriguezcerdeira et al., 2019). *C. albicans* is a dimorphic yeast that can interconvert from a yeast state to a hyphal state, commonly existing in mucosae and the digestive tract (Pizarrocerda and Cossart, 2006; Martin et al., 2018). Adherence of *C. albicans* to host cells, a prerequisite for infection, plays a significant role in pathogenesis, as it allows the establishment of a strong link to host cell surfaces and provides a focal point for infection by enabling persistence in harsh conditions (Pizarrocerda and Cossart, 2006; Martin et al., 2018). This high adherence is the main cause of fungal infection in human hosts, indicating that the highly infectious nature of this fungus may be related to its strong adherence capacity (Albrecht et al., 2006).

Numerous human infections are caused by biofilms of pathogenic microorganisms, especially fungi (Oshiro et al., 2019). Biofilm formation is initiated through adherence (Simon et al., 2018). Biofilms are complex microbial communities organized in an extracellular polymeric matrix comprising proteins, polysaccharides and DNA (Simon et al., 2018). Biofilm formation is a major concern in the clinic, as biofilms allow microorganisms to become significantly more resistant to antifungal agents and host immune clearance (Holm et al., 2015). The adhesion and biofilm formation of *C. albicans* significantly increases the risk of fungal infections with possible lethal complications (Tan et al., 2019). Therefore, new anti-adherence and anti-biofilm agents and strategies for *C. albicans* are urgently needed.

Natural products have been used as traditional medicines since ancient times (Boufridi and Quinn, 2018). *Houttuynia cordata* is an edible vegetable with a fishy taste which is widely consumed in southwest China, and also a traditional Chinese herb for treating lung and skin infections caused by pathogenic bacteria and fungi (Lu et al., 2013). Recent studies have indicated that *Houttuynia cordata* and its active ingredients demonstrate cogent antibacterial, anti-inflammatory, antiviral and anti-lung cancer activities (Jiang et al., 2019). The main effective component of *Houttuynia cordata* is sodium houttuyfonate (SH) which has a fishy odor, and has been widely used for the treatment of purulent skin infections and respiratory tract infections in China (Jiang et al., 2019). SH is not only effective against gram positive bacteria (Ye et al., 2007), but is also effective against fungi, and can be used as a synergistic companion with FLZ against *C. albicans* (Huang et al., 2015; Shao et al., 2017). Because of the instability of the chemical structure of SH, its derivative, SNH, has been synthesized with improved stability, and it also has a fishy odor (Lu et al., 2013). SNH has been widely used in pharmaceutical and medical applications in China and demonstrates extensive biological and antimicrobial properties (Yang et al., 2016). Previous studies have shown that SNH can significantly inhibit a variety of bacteria through H_2_O_2_ stress (Wu et al., 2015; Yang et al., 2016). However, the antifungal mechanism of SNH remains unclear. Therefore, we performed an RNA-seq assay to investigate the transcriptome changes in *C. albicans* treated using SNH. The transcription of genes involved in H_2_O_2_ stress was not significantly changed by SNH in *C. albicans*, implying that the antifungal mechanism of SNH may not be similar to its antibacterial mechanisms.

Therefore, in the current study, we aim to investigate the effects of SNH on the adherence and biofilm formation of *C. albicans* both *in vitro* and *in vivo*, and the possible antifungal mechanisms of SNH.

## Materials and Methods

### Strain and Cultivation

*Candida albicans* SC5314 was a gift from Prof. Yuanying Jiang, School of Pharmacy, Second Military Medical University (Shanghai, China). *C. albicans* Z103, Z3044, Z1402, and Z1407 were purchased from the Clinical Laboratory of Anhui Provincial Hospital (Hefei, China). *C. albicans* was streaked from −80°C glycerol stocks onto Sabouraud’s agar (Hope Biotech Co., Qingdao, China) plates. The strain was routinely inoculated for three generations in Sabouraud’s agar plates, a single colony was chosen for each inoculation, and then this was activated and propagated in liquid Sabouraud medium (Hope Biotech Co., Qingdao, China) at 37°C for 12–16 h until the strain reached the exponential growth phase. The revived *Candida* cells were pooled through centrifugation at 3000 g. After washing twice using sterile PBS (Leagene, Beijing, China), the *Candida* cells were resuspended in RPMI-1640 medium (Invitrogen, Carlsbad, CA, United States) and adjusted to a defined cell density using a hemocytometer prior to subsequent tests.

### Determination of Minimum Inhibitory Concentrations of 80% (MIC_80_)

Sodium new houttuyfonate, (HPLC ≥ 98%) was purchased from Shanghai Yuanye Bio-Technology Co., Ltd., and the production batch number was A23A9E59436. The *C. albicans* was diluted to 2 × 10^3^ CFU/mL. The minimum inhibitory concentration of 80% (MIC_80_) of SNH was determined in a 96-well flat-bottomed microplate (Corning, NA, United States) using the microdilution method. The SNH was serially two-fold diluted in a range of 1024–32 μg/mL. The fungal cells were incubated with the drugs used at 37°C for 24 h, and the OD value of each well was measured at 620 nm using a microplate reader. The value of the control well was controlled at approximately 0.2, and compared with the control well, the drug concentration in the lowest concentration well where the OD value decreased by more than 80% represented the MIC_80_ (the drug concentration when fungal growth was inhibited by 80%). The above experiment was repeated three times in parallel. It is accepted that when the MIC_80_ value can be accurately repeated or with only one concentration difference, then the higher concentration is used as the MIC_80_ value; when the MIC_80_ value differs by more than two concentrations, a re-test is needed until the requirements are met (Barchiesi et al., 1994; Pfaller and Barry, 1994).

### Determination of Minimum Fungicidal Concentrations (MFC)

To determine the minimum fungicidal concentration (MFC), 20 μL of medium was removed from the 96-well plate for MIC detection and diluted 100-fold in PBS, and added to a solid sand agar plate, spread evenly, and then placed in a 37°C incubator for 24 h, before counting the number of colonies (Silva et al., 2019). The MFC was determined as the lowest drug concentration such that fewer than three fungal colonies were observed after a 24 h incubation at 35°C. The MFC determinations were repeated three times.

### Initial Adhesive Growth Curve

The *C. albicans* solution was diluted to 2 × 10^6^ CFU/mL. After 0, 2 and 4 h of incubation, the fungal solution for each group in each time period was placed on a mixing shaker to fully shake it, until the cells adhering to the bottom of the tube were completely resuspended in the culture medium. Immediately, 100 μL of the suspension containing *Candida* for each group was serially diluted 1000-fold in PBS. The diluted suspension containing *Candida* was placed on the mixing shaker again, and fully shaken and mixed at the same time and frequency. A total of 100 μL of the 1000-fold diluted suspension was then evenly plated on Sabouraud’s agar in triplicate for a 24 h incubation at 37°C for yeast cell counting.

### XTT Reduction Assays

*Candida albicans* cell culture medium (100 μL; 2 × 10^6^ CFU/mL) was co-incubated with the same volumes of SNH or FLZ in a 96-well flat-bottomed microplate at 37°C for 2 h and 4 h (for adherence) or 24 h (for biofilms). The XTT salt [2,3-bis(2-methoxy-4-nitro-5-sulfophenyl)2H-tetrazolium-5-carboxanilide sodium salt] (Shanghai Yuanye Bio-Technology Co., Ltd., Shanghai, China) was dissolved in Ringer’s solution at a final concentration of 0.5 mg/mL. Immediately before each assay, a menadione (Shanghai Dibai Biotechnology Co., Ltd., Shanghai, China) solution was prepared at a final concentration of 0.4 mM and filter-sterilized. The XTT solution was mixed with the menadione solution at a ratio of 20:1 (v/v). Each well was washed twice with 200 μL of sterile PBS to remove planktonic cells, and finally 50 μL of newly prepared XTT solution was added to each well (Manoharan et al., 2017a; Rossoni et al., 2019). All well plates were incubated for 30 min in the dark, and colorimetric changes in the solutions were measured using a microplate reader (Thermo Labserv K3, Beijing, China) at 492 nm.

### Inverted Microscope

*Candida albicans* cell culture medium (1 mL; 2 × 10^6^ CFU/mL) was co-incubated with the same volumes of SNH or FLZ in a 6-well flat-bottomed microplate at 37°C for 4 h (for yeast) and 6 h (for hyphae). After that, all plates were observed using a high-power lens (×400) and photographed using the OLYMPUS IX51 (Tokyo, Japan).

### Quantitative Determination of Biofilms Using Crystal Violet

As previously reported (Manoharan et al., 2017b), *C. albicans* was inoculated into 96-well plates for a static biofilm formation assay. Overnight cultures of *C. albicans* strains were inoculated into Potato Dextrose Broth (PDB, Hangwei, Hangzhou, China) at a 600 nm initial turbidity of 0.05 (total volume of 200 μL), with or without essential oils or different concentrations of its main components (0.0005 to 0.05%, incubate under v/v or w/v) for 24 h, without shaking, at 37°C. The samples were washed twice using sterile PBS to remove all non-adherent planktonic cells, and then stained with crystal violet (Macklin, Shanghai, China) for 20 min, washed gently with sterile PBS twice and stained with crystal violet with 95% ethanol to quantify biofilm formation. The absorbance of each well was measured at a wavelength of 570 nm, and the results were calculated by averaging the data.

### Crystal Violet Staining

As previously reported (Manoharan et al., 2017c), slides with an area of 1 cm^2^ were soaked in a 75% ethanol solution for 24 h, washed with sterile PBS, and placed in a 6-well plate. *C. albicans* cell culture medium (1 mL; 2 × 10^6^ CFU/mL) was co-incubated with the same volumes of SNH or FLZ in a 6-well flat-bottomed microplate at 37°C for 24 h (for biofilms). After that, the upper layer medium was discarded and rinsed with sterile PBS three times, stained with crystal violet solution, observed using a high-power lens (×400) and photographed using an OLYMPUS BX51 (Tokyo, Japan).

### Confocal Laser Scanning Microscopy (CLSM)

*Candida albicans* cell culture medium (500 μL; 2 × 10^6^ CFU/mL) was co-incubated with the same volumes of SNH or FLZ in a 15mm glass-bottom confocal dish (Biosharp, Hefei, China) at 37°C for 24 h. After that, the upper layer medium was discarded and the dish was rinsed gently with PBS twice to remove planktonic fungi. Acridine orange dye solution (1 mg/mL, Yanye, Shanghai, China) was diluted to a final concentration of 0.01% in PBS, and then 200 μL of 0.01% acridine orange dye solution was added into the confocal dish and incubated for 30 min in the dark. Finally, a photograph was obtained using a camera mounted on a laser scanning confocal microscope at an excitation wavelength of 488 nm, and the AOD of the fluorescence in the photo was analyzed using Image J.

### Scanning Electron Microscopy (SEM)

Slides with an area of 1 cm^2^ were soaked in a 75% ethanol solution for 24 h, washed with sterile PBS, and placed in a 6-well plate. *C. albicans* cell culture medium (1 mL; 2 × 10^6^ CFU/mL) was co-incubated with the same volumes of SNH or FLZ in a 6-well flat-bottomed microplate at 37°C for 24 h (for biofilm). The slides were rinsed gently with PBS twice to remove planktonic fungi, and then placed in pre-chilled 2.5% glutaraldehyde solution and fixed for 2 h in the dark. The slides were then placed in an ethanol solution for gradient dehydration (30%, 10 min; 70%, 10 min; 90%, 10 min; 100%, 10 min), taken out and place in a ventilated and dry place to dry overnight. The glass slides were stuck in a metal plate using carbon tape and placed in a vacuum gold-plating machine for vacuum and gold plating. After the sample was prepared, it was placed into a Schottky Field Emission Scanning Electron Microscope (GeminiSEM 500, Germany) for observation (×500) and to acquire images.

### An Assessment of Synergy Between SNH and Several Antifungal Agents

Four antifungal agents were investigated: FLZ (HPLC ≥ 98%), BBR (HPLC ≥ 98%), CAS (HPLC ≥ 98%), and ITZ (HPLC ≥ 98%), which were purchased from Shanghai Yuanye Bio-Technology Co., Ltd. First, we tested the MIC_80_ concentration of these four antifungal agents alone against *Candida albicans* SC5314 through 96 well microplate experiments. After that, we combined these four antifungal agents with SNH separately, and conducted synergistic MIC_80_ experiments through a checkerboard microdilution method; that is, the two drugs were used in combination on a 96-well plate in a two-dimensional checkerboard longitudinal (A to H) and horizontal (2 to 11) two-fold dilution in two directions, respectively. For example, for the combined use of SNH and FLZ, the final concentration of SNH was controlled at 2–256 μg/mL, and the final concentration of FLZ was controlled at 0.005–4 μg/mL. The final concentration ranges of the other antifungal agents used in combination with SNH were as follows: the final concentration of BBR was controlled at 0.125–64 μg/mL, the final concentration of CAS was controlled at 0.005–4 μg/mL, and the final concentration of ITZ was controlled at 0.01–8 μg/mL.

Evaluation of the effect of combination medicine: The FICI is the main parameter for evaluating the interaction mode of two drugs. The FIC is the ratio of the MIC required for each drug when combined for antimicrobial activity and the MIC when used alone. FICI is equal to the sum of the FIC of the two drugs. This experiment used the latest standards of international journals: the interaction between the two drugs is determined to be synergistic when FICI ≤ 0.5, it is an irrelevant effect when 0.5 < FICI ≤ 4, and when FICI > 4, the two drugs produce an antagonistic effect.

We also used CLSM to observe the effect of SNH combined with several antifungal agents on a *Candida albicans* SC5314 biofilm, and set up a Control group, a MIC FLZ group, a MIC BBR group, a MIC CAS group, a MIC ITZ group and the above four drugs combined with MIC SNH group. The analysis method is consistent with the CLSM method described previously, and the AOD of the fluorescence in the photo was analyzed using Image J.

### Eukaryotic Transcriptome Sequencing

*Candida albicans* cells were harvested by centrifugation and the pellet was flash-frozen in liquid nitrogen. (1) Total RNA was extracted from the tissue using TRIzol^®^ Reagent (Plant RNA Purification Reagent for plant tissue) according the manufacturer’s instructions (Invitrogen) and genomic DNA was removed using DNase I (TaKara). Then RNA quality was determined by 2100 Bioanalyser (Agilent) and quantified using the ND-2000 (NanoDrop Technologies). Only high-quality RNA sample (OD260/280 = 1.8∼2.2, OD260/230 ≥ 2.0, RIN ≥ 6.5, 28S:18S ≥ 1.0, > 2 μg) was used to construct sequencing library. (2) RNA-seq transcriptome librariy was prepared following TruSeqTM RNA sample preparation Kit from Illumina (San Diego, CA, United States) using 1 μg of total RNA. Shortly, messenger RNA was isolated according to polyA selection method by oligo(dT) beads and then fragmented by fragmentation buffer firstly. Secondly double-stranded cDNA was synthesized using a SuperScript double-stranded cDNA synthesis kit (Invitrogen, CA, United States) with random hexamer primers (Illumina). Then the synthesized cDNA was subjected to end-repair, phosphorylation and “A” base addition according to Illumina’s library construction protocol. Libraries were size selected for cDNA target fragments of 200–300 bp on 2% Low Range Ultra Agarose followed by PCR amplified using Phusion DNA polymerase (NEB) for 15 PCR cycles. After quantified by TBS380, paired-end RNA-seq sequencing library was sequenced with the Illumina HiSeq xten/NovaSeq 6000 sequencer (2 × 150 bp read length). The Illumina platform converts the sequenced image signals into textual signals via CASAVA Base Calling and stores them in raw data in fastq format, and performs sequencing-related quality assessments on the raw sequencing data for each sample. (3) The raw paired end reads were trimmed and quality controlled by SeqPrep^1^ and Sickle^2^ with default parameters. Then clean reads were separately aligned to reference genome with orientation mode using TopHat^3^ (Trapnell et al., 2009) software. The mapping criteria of bowtie was as follows: sequencing reads should be uniquely matched to the genome allowing up to 2 mismatches, without insertions or deletions. Then the region of gene were expanded following depths of sites and the operon was obtained. In addition, the whole genome was split into multiple 15kbp windows that share 5kbp. New transcribed regions were defined as more than 2 consecutive windows without overlapped region of gene, where at least 2 reads mapped per window in the same orientation. (4) To identify DEGs (differential expression genes) between two different samples, the expression level of each transcript was calculated according to the fragments per kilobase of exon per million mapped reads (FRKM) method. RSEM^4^ (Li and Dewey, 2011) was used to quantify gene abundances. R statistical package software EdgeR (Empirical analysis of Digital Gene Expression in R^5^, Robinson et al., 2010) was utilized for differential expression analysis. In addition, functional-enrichment analysis including GO and Kyoto Encyclopedia of Genes and Genomes (KEGG) were performed to identify which DEGs were significantly enriched in GO terms and metabolic pathways at Bonferroni-corrected *P*-value ≤ 0.05 compared with the whole-transcriptome background. GO functional enrichment and KEGG pathway analysis were carried out by Goatools^6^ and KOBAS^7^ (Xie et al., 2011). (5) The TopHat-Cufflinks pipeline was used to predict gene isoforms from our RNA-seq data. In TopHat^8^ (Trapnell et al., 2009), the option “min-isoform-fraction” was disabled, instead “coverage-search,” “butterfly search,” and “microexon-search” were used. The expected fragment length was set to 200 bp and the “small-anchor-fraction” was set to 0.08, which requires at least 8 bp on each side of an exon junction for our 100-bp RNA-seq data. Cuffcompare was used to compare and merge the reference annotation and the isoform predictions. (6) All the alternative splice events that occurred in our sample were identified by using recently releases program Multivariate Analysis of Transcript Splicing (MATS^9^) (Shen et al., 2012). Only the isoforms that were similar to the reference or comprised novel splice junctions were considered, and the splicing differences were detected as exon inclusion, exclusion, alternative 5′ AGATCGGAAGAGCACACGTC, 3′ AGATCGGAAGAGCGTCGTGT, and intron retention events. The high-quality sequencing data were analyzed on the free online platform of Majorbio I-Sanger Cloud Platform^10^. The transcriptomic data have been deposited into the SRA database under the accession numbers of PRJNA544616 (https://www.ncbi.nlm.nih.gov/sra/PRJNA544616).

### Quantitative RT-PCR

Firstly, a 1 × 10^6^ CFU/mL fungal culture was incubated for 4 h (for adherence) or 24 h (for biofilms) at 37°C. The fungal cells were collected through centrifugation for 3 min at 12700 *g*, and then total RNA samples were extracted in accordance with the manufacturer’s instructions for the MagExtractor-RNA kit (Toyobo, Tokyo, Japan). Extracted total RNA (6 μL) was mixed with 2 μL of 4 × DNA Master I (containing gDNA remover) and 2 μL 5RT-Master Mix II through instant centrifugation at 3000 *g*, and reverse-transcribed into cDNA as recommended by the instructions of the ReverTra Ace qPCR RT Master Mix with gDNA Remover kit (Toyobo, Tokyo, Japan), with procedures as follows: pre-denature RNA in GeneTest series gene amplifier (BIO-GENER, Hangzhou, China) at 65°C for 5 min, 4°C for 1 min, and then at 37°C for 15 min, 50°C for 5 min, 98°C for 5 min, and 4°C for 1 min to finally generate cDNA. The prepared cDNA was diluted 10-fold prior to use for semi-quantitative PCR and qRT-PCR. Primers for *C. albicans* (Table 1) were designed using Primer Premier 5.0 and synthesized by Sangon Biotech. The qRT-PCR mixture (25 μL) was freshly prepared containing 12.5 μL of SYBR green fluorescent dyes, 1 μL of PCR forward primer, 1 μL of PCR reverse primer, 0.5 μL of cDNA, and 10 μL of DEPC treated water. The qRT-PCR process was performed using an ABI7000 fluorescence quantitative PCR system with the following cycles: 95°C for 60 s for pre-denaturation alone with 95°C for 15 s, 50°C for 15 s, and 72°C for 45 s for a total of 40 cycles. All data were normalized to the housekeeping gene β-actin as the internal reference gene. The relative target-gene expression was calculated as a fold change of the 2^–ΔΔ*Ct*^ value, in which ΔCt = Ct target gene – Ct internal reference genes, as previously described (Livak and Schmittgen, 2001).

**TABLE 1:** Primers for qRT-PCR.

**Oligo Name**	**Sequence (5′ to 3′)**	**Length (bp)**
β-*actin*-Forward	ACC GAA GCT CCA ATG AAT CC	20
β-*actin*-Reverse	CCG GTG GTT CTA CCA GAA GAG	21
*ALS1*-Forward	GGA TCT GTT ACT GGT GGA GCT GTT G	25
*ALS1*-Reverse	ATG TGT TGG TTG AAG GTG AGG ATG AG	26
*ALA1*-Forward	GGC TAC GTG TTA CAC ACT GGT	21
*ALA1*-Reverse	TCA ACG CCA TCT CCA AGG AC	20
*ALS3*-Forward	CAA CTT GGG TTA TTG AAA CAA AAA CA	26
*ALS3-*Reverse	AGA AAC AGA AAC CCA AGA ACA ACC T	25
*EAP1*-Forward	ATC TAC CTC CTA CAC GAC TGA CAC TG	26
*EAP1*-Reverse	TGT ATG AGA ACA AGA ACC GCC ATC AC	26
*RAS1*-Forward	GAG GTG GTG GTG TTG GTA	18
*RAS1*-Reverse	TTC TTC TTG TCC AGC AGT ATC	21
*EFG1*-Forward	ATT GAG ATG TTG CGG CAG GAT AC	23
*EFG1*-Reverse	ACT GGA CAG ACA GCA GGA C	19
*HWP1*-Forward	ACA GGT AGA CGG TCA AGG	18
*HWP1*-Reverse	GGG TAA TCA TCA CAT GGT TC	20
*TEC1*-Forward	AGG TTC CCT GGT TTA AGT G	19
*TEC1*-Reverse	ACT GGT ATG TGT GGG TGA T	19
*YWP1*- Forward	CTG ATA TTC GTA ATG CTG GTA AAG TG	26
*YWP1*- Reverse	GGA GTT TCA CCC ATT AAT CTT CTT C	25
*RHD1*-Forward	TTA GAG AAA TGT GGC TGT GGT G	22
*RHD1*-Reverse	TCA CAT AAC CCT TTA TCA GGC C	22

### Semi-Quantitative PCR

The preparation steps for the cDNA are the same as those described previously. The primer sequences used in the semi-quantitative PCR are shown in Table 1. The PCR mixture (25 μL) was freshly prepared containing 12.5 μL of 2 × EasyTaq^®^ PCR SuperMix, 0.5 μL of PCR forward primer, 0.5 μL of PCR reverse primer, 0.5 μL of cDNA, and 11 μL of DEPC treated water. PCR conditions were as follows: 94°C for 5 min, 35 cycles at 94°C for 30 s, 50°C for 30 s, 72°C for 30 s, with a final extension at 72°C for 5 min. The PCR process was performed using a GeneTest series gene amplifier (BIO-GENER, Hangzhou, China). PCR products were analyzed using a 1.5% agarose gel and compared with a D2000 plus DNA Ladder (Solarbio, Beijing, China). The horizontal electrophoresis conditions were 110 v, 80 mA, 20 min, and the agarose gel was placed into a JY04S-3E imaging analysis system (Junyi, Beijing, China) for gel imaging. The brightness of the PCR products was observed, and it was proportional to the target gene expression.

### Detection of cAMP Levels

*Candida albicans* cell culture medium (1 mL; 2 × 10^6^ CFU/mL) was co-incubated with the same volumes of SNH or FLZ in a 6-well flat-bottomed microplate at 37°C for 2 h and 4 h (for adherence) or 24 h (for biofilms). The *Candida* cells were scraped using a cell scraper, suspended in medium, and centrifuged for 3 min at 12700 *g*. The supernatant was analyzed for cAMP in accordance with the manufacturer’s protocol, and the cAMP concentration of each group was examined using a microplate reader (Thermo Labserv K3, Beijing, China) and ELISA kits purchased from RUIXIN Biotech (Quanzhou, China).

### *In vivo* Evaluation of Antifungal Efficacy of SNH Against *C. albicans*

Old *Galleria mellonella* larvae were purchased from Tianjin Huiyude Biological Technology Co., Ltd., each weighing 0.2–0.3 g, and they were approximately 2–3 cm in length. They were stored in a refrigerator at 4°C until use. The larvae were placed on an ultra-clean bench, activated at room temperature for 30 min, and then the *in vivo* injection experiment was performed.

Preparation of *Candida* injection: a single colony was chosen on the Sabouraud’s agar plate and inoculated into liquid Sabouraud medium, which was placed on a shaker at 220 rpm, at 37°C, for 12 h, and then the cells were collected, the upper medium was removed, and the cells were resuspend in PBS. The cell concentration was adjusted to 8 × 10^6^ CFU/mL, 10 μL of *Candida* solution was injected into the right anterior side of the second section of the stomach and foot. Larvae were inoculated with 8 × 10^4^ yeast cells per 10 μL into the hemolymph, as described previously (Bergin et al., 2006; Harding et al., 2013; Manoharan et al., 2017b).

All drugs were dissolved in PBS without any cosolvent to avoid the toxic effects of cosolvents for insects. The drugs were sonicated for a few seconds, and then placed in a75°C water bath for 20 min. During this period, the drug solutions were shaken and mixed continuously until the drug was completely dissolved and diluted with PBS to 512 μg/mL, 256 μg/mL, and 128 μg/mL (SNH), and 128 μg/mL and 64 μg/mL (FLZ).

*Galleria mellonella* larvae (*n* = 20 larvae per group) were grouped as follows: PBS group, infection (*C. albicans* 8 × 10^4^ CFU/larvae) group, infection + SNH 512 μg/mL group, infection + SNH 256 μg/mL group, infection + SNH 128 μg/mL group, infection + FLZ 128 μg/mL group, infection + FLZ 64 μg/mL group, uninfected + SNH 512 μg/mL group, uninfected + SNH 256 μg/mL group, uninfected + SNH 128 μg/mL group, uninfected + FLZ 128 μg/mL group and uninfected + FLZ 64 μg/mL group. Larvae in the infected group were infected with PBS containing *C. albicans* SC5314 strains (LD_50_ = 8 × 10^4^ CFU/larvae); drugs groups were administered 1 h post-infection with 10 μL of SNH or FLZ. The PBS group and uninfected group were injected with sterile PBS. The changes in the larvae body were observed every 12 h. If the larvae body was melanized and there was no turning movement, the larvae body was considered to be dead. The larvae were observed continuously for 84 h and the number of deaths was recorded to determine the survival rate of each group. In view of studies that show that the LD_50_ of SNH is 119.14 mg/kg (Jiang et al., 2006) and the LD_100_ of SNH is 200 mg/kg (Lou et al., 2012), our research group selected the dose range 1024–32 μg/mL for SH in previous experiments (Wang et al., 2019; Zhao et al., 2019). Therefore, the safety range of SNH is large, and it has no obvious cytotoxicity.

### Statistical Analysis

All experiments were repeated at least three times. Data represent biological replicates. Data meet the assumptions of the statistical tests described for each Figure. Results are expressed as the mean ± SD. Differences between experimental groups were assessed for significance using a two-tailed unpaired Student’s *t*-test with GraphPad Prism 5 software. The ^∗^*p* < 0.05, ^∗∗^*p* < 0.01, and ^∗∗∗^*p* < 0.001 levels were considered to indicate statistical significance.

## Results

### Determination of MIC_80_ and MFC

**FIGURE 1:**
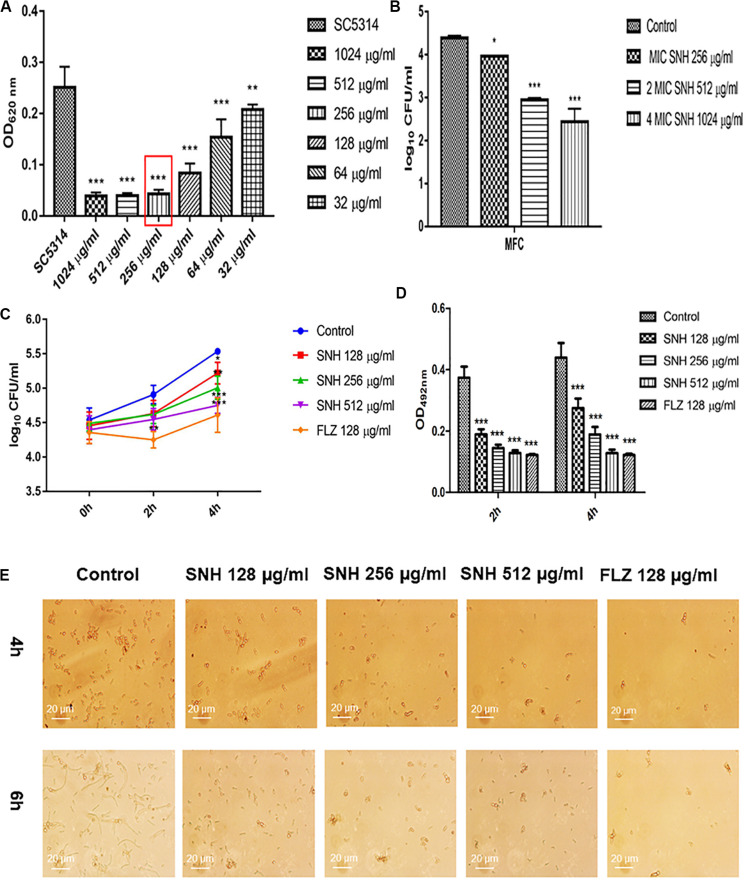
**(A)** Determination of MIC_80_ of SNH on *C. albicans* SC5314 by microdilution method. The drug-free strain-containing medium was set as the control. Data are shown as mean ± SD. ***p < 0.001 and **p < 0.01, compared with the control. **(B)** Determination of MFC of SNH on *C. albicans* SC5314 by plate count. The drug-free strain-containing medium was set as the control. Data are shown as mean ± SD. ***p < 0.001 and *p < 0.05, compared with the control. **(C)** Effect of SNH on CFU of adhesive cells of *C. albicans* SC5314. The initial adhesive growth curve measurements were taken for the first 4h. The drug-free strain-containing medium was set as the control. Data are shown as mean ± SD. *p < 0.05, compared with the control. **(D)** Effect of SNH on adherence metabolic activity in *C. albicans* SC5314 by the XTT reduction assay. The drug-free strain-containing medium was set as the control. Data are shown as mean ± SD. ***p < 0.001, compared with the control. **(E)** Observation of morphological changes of *C. albicans* yeast (4 h) and hypha (6 h) by inverted microscope (×400). The initial inoculum was newly prepared at 2 × 10^6^ CFU/mL. The drug-free strain-containing medium was set as the control.

Using a microplate reader, we found (Figure 1A) that the cell growth ratio of the 256 μg/mL SNH group to the control group was 17.15%; that is, 82.85% of *Candida* growth was inhibited within 24 h. The value of the control well was controlled at approximately 0.2, and compared with the control well, the drug concentration in the lowest concentration well where the OD value decreased by more than 80% was the MIC_80_ (the drug concentration when fungal growth was inhibited by 80%). Therefore, the MIC_80_ of SNH in *C. albicans* SC5314 was determined to be 256 μg/mL. The 64 μg/mL (1/4 MIC_80_) dose group had a poor fungal inhibitory effect and had no practical clinical significance, and the 1024 μg/mL (4 MIC_80_) dose group had fungal inhibitory effects that were too strong, which does not conform to the principle of using the lowest dose within the effective dose range. Therefore, the 64 μg/mL and 1024 μg/mL dose groups were not investigated further. We observed the effects of 128 μg/mL (1/2 MIC_80_), 256 μg/mL (1 MIC_80_), and 512 μg/mL (2 MIC_80_) SNH, and 128 μg/mL FLZ on the adherence and biofilm formation of *C. albicans*. In addition, through MFC detection, it was found that with SNH at MIC, 2 MIC and 4 MIC for 24 h the number of colonies was greater than three (Figure 1B), indicating that SNH has no fungicidal effect on *C. albicans* SC5314, and only inhibits fungal action.

### SNH Inhibits *C. albicans* Adhesion and Hypha Growth

Through a CFU plate count (Figure 1C), we found that SNH treatment for 0 h resulted in no significant difference compared with the control group. After 2 h of SNH treatment, the CFU counts in the SNH groups were less than those in the control group, but the difference was not statistically significant. After 4 h of SNH treatment, the CFU counts in the SNH groups were significantly less than those in the control group, and the difference was statistically significant. The *p*-Value for the SNH 512 μg/mL group was <0.001, the *p*-Value for the SNH 256 μg/mL group was <0.01, and the *p*-Value for the SNH 128 μg/mL group was <0.05. This shows that SNH exerts the best anti-fungal effect by 4 h of initial adhesion. In addition, through an XTT assay, we found that the addition of SNH to *C. albicans* after a 2 and 4 h incubation can significantly reduce adherence activity (Figure 1D) in a concentration-dependent manner. The *p*-Value of the SNH groups at 2 h and 4 h were <0.001. Therefore, the results show that SNH can effectively inhibit the initial adhesion activity of *C. albicans*. The inverted microscope results (Figure 1E) indicate that SNH and FLZ treatment are similar and can effectively reduce cell adhesion in *C. albicans* when administered for 4 h. In addition, we also found that the control group demonstrated obvious slender hyphae at 6 h, while the hyphae of *C. albicans* were effectively inhibited after SNH treatment, whereby only a few yeast-like cells appeared, and no obvious hyphae were observed (Figure 1E). This result suggests that SNH can effectively inhibit the transition of yeast to hyphae in *C. albicans*, thereby providing the possibility of biofilm inhibition.

### SNH Affects the Biofilm Formation of *C. albicans*

**FIGURE 2:**
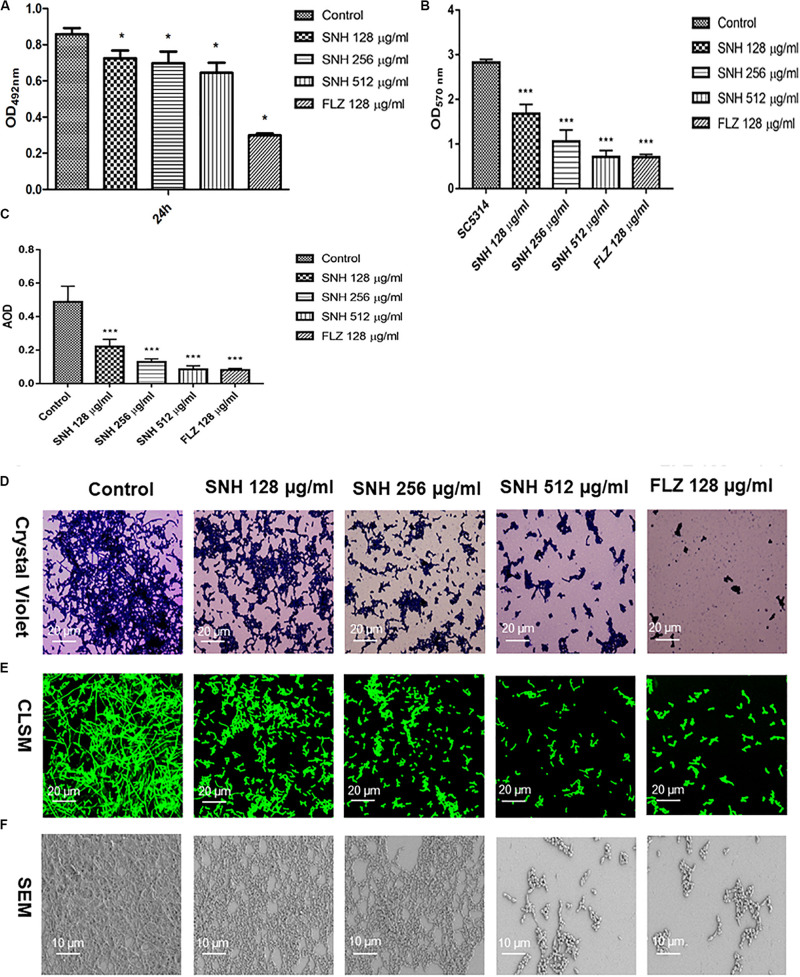
**(A)** Effect of SNH on biofilm metabolic activity in *C. albicans* SC5314 by the XTT reduction assay. The drug-free strain-containing medium was set as the control. Data are shown as mean ± SD. *p < 0.05, compared with the control. **(B)** Quantitative determination of *C. albicans* SC5314 biofilm by crystal violet. The drug-free strain-containing medium was set as the control. Data are shown as mean ± SD. ***p < 0.001, compared with the control. **(C)** Analyze the AOD (AOD) of CLSM fluorescent *C. albicans* SC5314 biofilm by Image J. The drug-free strain-containing medium was set as the control. Data are shown as mean ± SD. ***p < 0.001, compared with the control. **(D)** Crystal Violet Staining of *C. albicans* SC5314 biofilm by optical microscope (×400). **(E)** Observation of morphological changes of *C. albicans* SC5314 biofilm by CLSM (×400). **(F)** Observation of morphological changes of *C. albicans* SC5314 biofilm by SEM (×500). The initial inoculum was newly prepared at 2 × 10^6^ CFU/mL. The drug-free strain-containing medium was set as the control.

Through an XTT assay (Figure 2A), we found that at 24 h, compared with the control group, SNH treatment of *C. albicans* significantly reduced the biofilm OD_492_ value in a concentration-dependent manner. Each SNH group demonstrated effectively inhibited biofilm metabolic activity (*p* < 0.05). In addition, through the crystal violet quantitative biofilm experiment (Figure 2B), it was also found that SNH can effectively inhibit biofilm dispersion and biofilm volume (*p* < 0.001). Among the groups, the SNH 512 μg/mL group inhibited biofilm growth by 74.91%, the SNH 256 μg/mL group inhibited biofilm growth by 62.51%, and the SNH 128 μg/mL group inhibited biofilm growth by 40.63%. We also observed the growth and morphological changes in *C. albicans* biofilms after SNH treatment through crystal violet staining, CLSM, and SEM (Figures 2D–F). The images showed that the control group demonstrated a large and thick biofilm at 24 h. SNH inhibited the formation of biofilms at 24 h, but single fungal bodies and a small biofilm structure was observed, and so it appears that SNH inhibits the formation of large biofilm structures. In addition, the CLSM analysis (Figure 2C) also showed that SNH can effectively inhibit biofilm formation (*p* < 0.001). In summary, SNH has the potential to inhibit *C. albicans* biofilms.

### SNH Inhibits Biofilms of Several Clinical Strains of *C. albicans*

**FIGURE 3:**
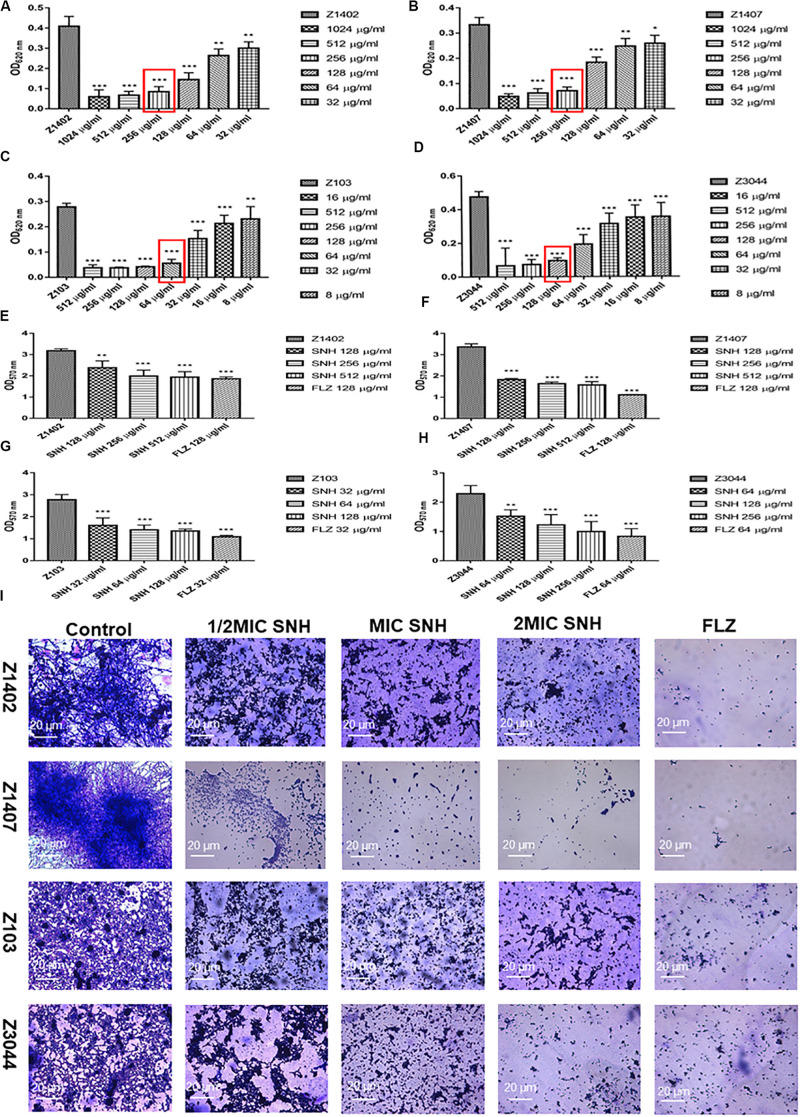
**(A–D)** Determination of MIC_80_ of SNH on *C. albicans* Z1402, Z1407, Z103, and Z3044 by microdilution method, respectively. The drug-free strain-containing medium was set as the control. Data are shown as mean ± SD. ***p < 0.001, **p < 0.01, and *p < 0.05, compared with the control. **(E−H)** Quantitative determination of *C. albicans* Z1402, Z1407, Z103 and Z3044 biofilm by crystal violet, respectively. The drug-free strain-containing medium was set as the control. Data are shown as mean ± SD. ***p < 0.001 and **p < 0.01, compared with the control. **(I)** Crystal Violet Staining of *C. albicans* Z1402, Z1407, Z103, and Z3044 biofilm by optical microscope (×400), respectively. The initial inoculum was newly prepared at 2 × 10^6^ CFU/mL. The drug-free strain-containing medium was set as the control.

To further investigate the potential of SNH against *C. albicans* biofilms, we selected four clinical strains of *C. albicans*, Z1402, Z1407, Z103, and Z3044 for these experiments. First, the MIC_80_ of SNH in clinical strains of *C. albicans* was detected through a 96-well microdilution method. Interestingly, the MIC_80_ of SNH in clinical strains was better than that in standard strains, whereby the MIC_80_ of SNH in clinical strains decreased 1–2-fold compared with that in standard strains. The MIC_80_ of SNH in Z103 was 64 μg/mL (Figure 3C) and the MIC_80_ of SNH in Z3044 was 128 μg/mL (Figure 3D). Moreover, the MIC_80_ of SNH in both Z1402 and Z1407 was 256 μg/mL (Figures 3A,B), which was the same as that in SC5314. We further tested the biofilm dispersion and biofilm quantity in four clinical strains treated using SNH through a crystal violet quantitative biofilm experiment. Each clinical strain was established in 2 MIC SNH, MIC SNH and 1/2 MIC SNH groups. It was found that the OD_570__nm_ of each SNH group was significantly lower than that of the control group (*p* < 0.01). The SNH 512 μg/mL group demonstrated 38.61% inhibition of biofilm growth of Z1402, the SNH 256 μg/mL group demonstrated 37.24% inhibition of biofilm growth of Z1402, and the SNH 128 μg/mL group demonstrated 25.06% inhibition of biofilm growth of Z1402 (Figure 3E). The SNH 512 μg/mL group demonstrated 53.08% inhibition of biofilm growth of Z1407, the SNH 256 μg/mL group demonstrated 51.41% inhibition of biofilm growth of Z1407, and the SNH 128 μg/mL group demonstrated 45.73% inhibition of biofilm growth of Z1407 (Figure 3F). The SNH 512 μg/mL group demonstrated 51.38% inhibition of biofilm growth of Z103, the SNH 256 μg/mL group demonstrated 49.52% inhibition of biofilm growth of Z103, and the SNH 128 μg/mL group demonstrated 42.03% inhibition of biofilm growth of Z103 (Figure 3G). The SNH 512 μg/mL group demonstrated 56.32% inhibition of biofilm growth of Z3044, the SNH 256 μg/mL group demonstrated 46.51% inhibition of biofilm growth of Z3044, and the SNH 128 μg/mL group demonstrated 33.49% inhibition of biofilm growth of Z3044 (Figure 3H). We also observed biofilm growth and morphological changes in four clinical *C. albicans* strains after SNH treatment through crystal violet staining (Figure 3I). The images show that the clinical strain control groups all demonstrated large and thick biofilms at 24 h. While SNH can inhibit biofilm formation at 24 h, small biofilm structures could be seen, and therefore it could be considered that SNH inhibits the formation of large biofilm structures of *C. albicans* clinical strains. In terms of the ability of 2 MIC SNH to inhibit biofilms, SNH at this concentration can reduce the biofilm quantities of Z3044, Z1407 and Z103 by more than 50%, but has a poor ability to inhibit the biofilms of Z1402. In terms of the ability of 1 MIC SNH to inhibit biofilms, SNH at this concentration can inhibit biofilms by more than 50% for Z1407. In terms of the ability of 1/2 MIC SNH to inhibit biofilms, SNH inhibition of the biofilms of clinical strains was weakened, and no group with more than 50% biofilm inhibition was observed. In general, the ability of SNH to inhibit the biofilms of *C. albicans* clinical strains was in the order: Z1407 > Z103 > Z3044 > Z1402.

### SNH Combined With Various Categories of Antifungal Against *C. albicans* Biofilm

**FIGURE 4:**
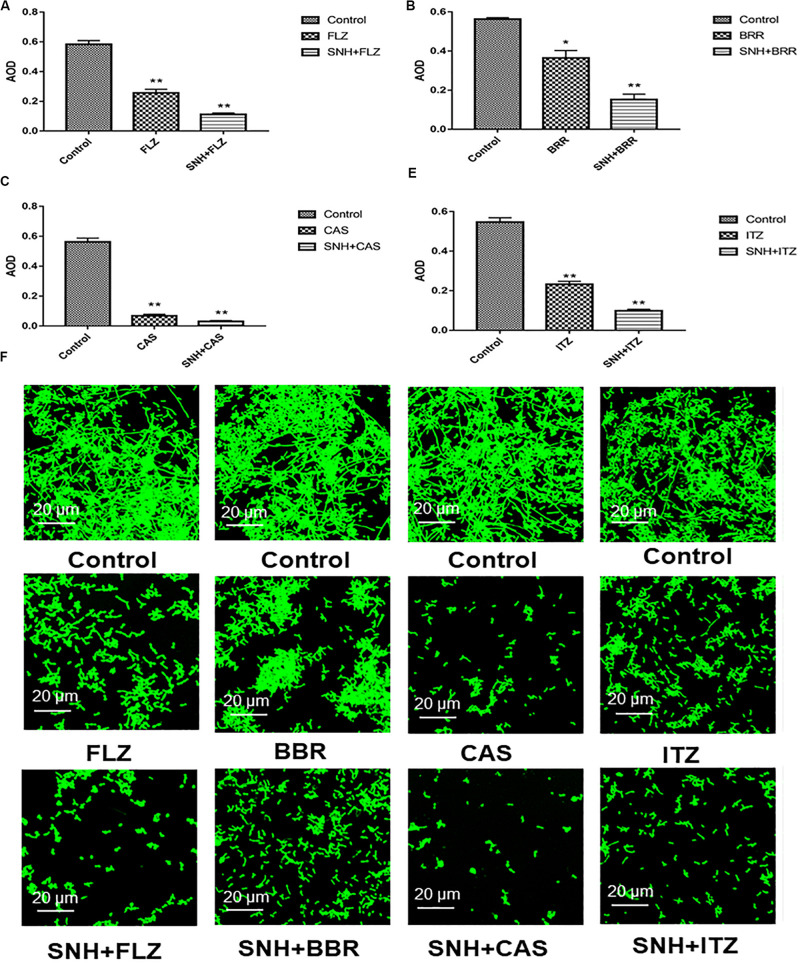
**(A–E)** Analyze the average optical density (AOD) of SNH and FLZ, BBR, CAS, ITZ on *C. albicans* SC5314 biofilm by Image J, respectively. The drug-free strain-containing medium was set as the control. Data are shown as mean ± SD. **p < 0.01 and *p < 0.05, compared with the control. **(F)** Observation of morphological changes of SNH and FLZ, BBR, CAS, ITZ on *C. albicans* SC5314 biofilm by CLSM (×400), respectively. The initial inoculum was newly prepared at 2 × 10^6^ CFU/mL. The drug-free strain-containing medium was set as the control.

Here, we investigated four antifungals, FLZ, BBR, CAS, and ITZ, combined with SNH to observe their ability to inhibit *C. albicans* SC5314 biofilms. First, we tested the MIC_80_ of the four antifungal drugs against *C. albicans* SC5314 through a microdilution method in 96-well plates, and then tested the MIC_80_ of the four antifungal drugs combined with SNH using a checkerboard microdilution method. The results are shown in Table 2. It was found that the FICI of SNH combined with FLZ, BBR, CAS, or ITZ was 0.27, 0.50, 0.27, and 0.50, respectively. These FICI values were all ≤0.05, which indicated that SNH had synergistic effects with these four antifungals. We further investigated the effect of SNH combined with the four antifungals against *C. albicans* biofilms through CLSM (Figure 4F), and analyzed the AOD value of the fluorescence images (Figures 4A–E). Here, we investigated the drugs alone and in combination with a 1 MIC dose for the experiments. The results showed that the four antifungals alone had anti-biofilm abilities, and minimal biofilm growth and yeast cells could be seen. However, when the four antifungals were combined with SNH, their anti-biofilm capabilities were strengthened, and compared with the control group, large-scale hyphal structures were completely inhibited, and only single yeast-like cells were observed. Moreover, compared with the control group, the anti-biofilm activity observed following treatment with SNH combined with the four antifungals was significantly enhanced (*p* < 0.01).

**TABLE 2:** Interaction of SNH and antifungals against *C. albicans* SC5314 by MIC_80_ of checkerboard microdilution assay.

	**MIC_80_ (μg/ml) alone**	**MIC_80_ (μg/ml) in combination**	**FIC index for combination**	**Mode of interaction**
SNH/FLZ	256/1	4/0.25	0.27	Syn
SNH/BBR	256/16	64/4	0.50	Syn
SNH/CAS	256/0.25	1/0.125	0.50	Syn
SNH/ITZ	256/8	4/2	0.27	Syn

*SNH, Sodium new houttuyfonate; FLZ, Fluconazole; BBR, Berberine chloride; CAS, Caspofungin; ITZ, Itraconazole; Syn, Synergism.*

### SNH Affects the Transcriptome of *C. albicans*

**FIGURE 5:**
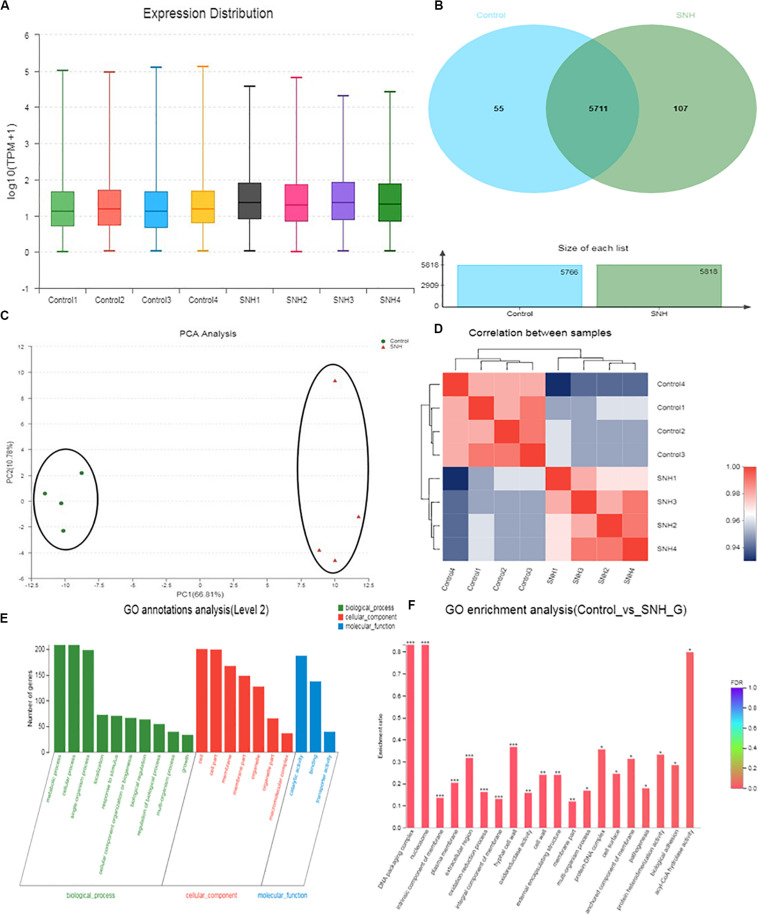
Effect of SNH on the transcriptome of *C. albicans* SC5314. **(A)** The abscissa is the sample name and the ordinate is log_10_ (TPM + 1). Each color in the figure represents a sample, and the horizontal line in the figure indicates the median of gene expression in the sample. **(B)** Venn diagram of the number of genes in the Control and SNH treatments, respectively. **(C)** The right and lower sides of the figure are sample names, and the left and upper sides are sample clustering, and the squares of different colors represent the correlation between the two samples in the principle component analysis (PCA). **(D)** After the sample is analyzed by dimensionality reduction, there are relative coordinate points on the principal component. The distance between each sample point represents the distance of the sample. The closer the distance is, the higher the similarity between samples. The horizontal axis represents the contribution of principal component 1 (PC1) to the differentiated samples in the two-dimensional map, and the vertical axis represents the contribution of principal component 2 (PC2) to the differentiated samples in the two-dimensional map. **(E)** The abscissa in the figure represents the secondary classification term of GO, the left ordinate indicates the percentage of genes or transcripts contained in the secondary classification, and the right ordinate indicates the genetic/transcription of the secondary classification. **(F)** The abscissa indicates the GO term, and the ordinate indicates the enrichment rate refers to the ratio of the number of genes/samples enriched in the GO term to the number of the annotation gene/background number. The color indicates the significance of the enrichment, and the redder the default color indicates that the GO term is significantly more enriched, with the FDR < 0.001 mark being ***, the FDR < 0.01 mark being **, and the FDR < 0.05 mark being *.

The results described above indicate that SNH can effectively inhibit adhesion and biofilm formation in *C. albicans*. To elucidate the underlying mechanism, we examined the transcriptomic changes in *C. albicans* under SNH treatment. As shown in Figure 5A, the total transcriptomes of the blank control group and the 256 μg/mL SNH group are basically consistent. As shown in Figure 5B, 5711 genes overlap in the two groups, with 55 and 107 genes uniquely expressed in the Control and SNH treatment groups, respectively. As shown in Figures 5C,D, we found that the expression levels in the blank control group and the 256 μg/mL SNH group were significantly clustered in two groups, while the blank control group and the 256 μg/mL SNH group had high intra-group correlation. As shown in the volcano map in Supplementary Figure S1, a total of 5873 genes are assembled in the two groups. Compared with the control group, the expression of 611 genes, including 361 down-regulated and 250 up-regulated genes, changed significantly in the SNH group with fold changes >2 and a *p*-Value < 0.05 (Supplementary Figure S1).

To gain insights into the biological pathways related to the 611 genes, hierarchical clustering (Supplementary Figure S2) of these genes was performed using Matlab to group the data into those genes increasing and decreasing in abundance following SNH treatment. GO enrichment results (Figures 5E,F) indicate that the genes generally down-regulated in SNH treatment are enriched in pathways associated with biological processes including regulation of biological process, growth, detoxification, response to stimulus, cellular processes, biological regulation, biological adhesion, cell aggregation, and cellular component biosynthesis including cell, cell part, membrane, membrane part and extracellular region, and molecular function including binding, catalytic activity and antioxidant activity. As shown in Supplementary Table S1, SNH can significantly down-regulate the expression of several genes, such as *RAS1*, *ALS3*, *and HWP1*, which are key to the pathogenesis of *C. albicans*. The three genes are involved in biological adhesion, cell aggregation and positive regulation of biological processes and also members of the Ras1-cAMP-Efg1 pathway.

### SNH Affects the Expression of Biofilm Related Genes and cAMP Production of the Ras1-cAMP-Efg1 Pathway

**FIGURE 6:**
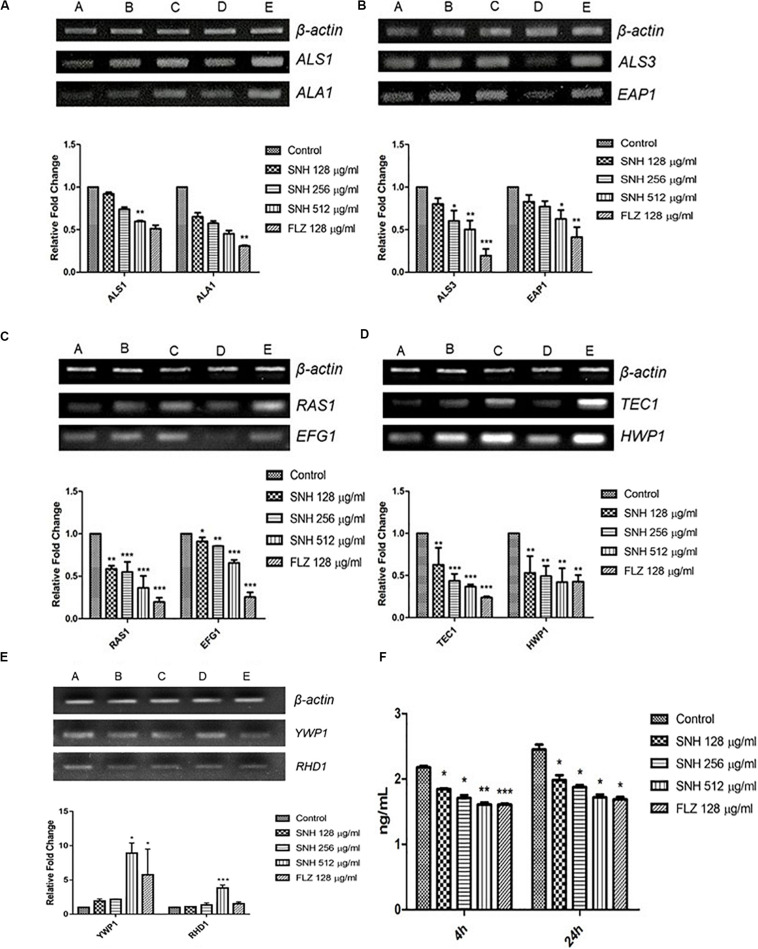
Expression changes of biofilm related genes and protein of Ras1-cAMP-Efg1 pathway by SNH treatment in *C. albicans* SC5314. **(A)** The expression levels of adhesion-related genes *ALA1* and *ALS1*. **(B)** The expression levels of biofilm-related genes *ALS3* and *EAP1*. **(C−D)** The expression levels of Ras1-cAMP-Efg1 pathway-related genes *RAS1, EFG1, TEC1*, and *HWP1.* **(E)** The expression of yeasts-related genes *YWP1* and *RHD1*. Gene expression results were detected by RT-PCR nucleic acid electrophoresis bands and qRT-PCR quantitative analysis. The drug-free strain-containing medium was set as the control. Data are shown as mean ± SD. (*A,B,C,D,E*) were respectively expressed as SNH 512 μg/mL group, SNH 256 μg/mL group, SNH 128 μg/mL group, and FLZ 128 μg/mL group, Control group. *p < 0.05, **p < 0.01, and ***p < 0.001 are calculated by comparing with the control group. **(F)** Effects of SNH on the production of cAMP protein of *C. albicans* SC5314. The drug-free strain-containing medium was set as the control. Data are shown as mean ± SD. *p < 0.05, **p < 0.01, and ***p < 0.001 are calculated by comparing with the control group.

To verify the transcriptome results described above, we examined several genes related to adhesion and biofilms, especially those related to the Ras1-cAMP-Efg1 pathway through the semi-quantitative PCR and qRT-PCR methods. In accordance with the transcriptome data, the expression of *ALS1*, *ALA1*, *ALS3*, *EAP1*, *RAS1*, *EFG1*, *HWP1*, and *TEC1* in this pathway are down-regulated after SNH treatment in a dose dependent manner (Figures 6A−D) and the expression of *YWP1* and *RHD1* are up-regulated after SNH treatment in a dose dependent manner (Figure 6E), which could explain the defects in adhesion, transition from yeast to hyphae and biofilm formation of *C. albicans* under SNH treatment. Among these, *ALS1* and *ALA1* are involved in the initial adhesion of *C. albicans*, and *ALS3* and *EAP1* are involved in the formation of *C. albicans* biofilms. Compared with the control group, SNH treatment significantly reduced the expression levels of the adhesion-related genes, *ALA1* and *ALS1* (Figure 6A). The *ALA1* gene in the SNH 512, 256, and 128 μg/mL groups was down-regulated by 1.91-fold, 1.65-fold, and 1.39-fold, respectively, and the *ALS1* gene in the SNH 512, 256, and 128 μg/mL groups was down-regulated by 1.65-fold, 1.39-fold, and 1.16-fold, respectively. Moreover, SNH reduced the expression levels of the biofilm-related genes *ALS3* and *EAP1* (Figure 6B); specifically, the *ALS3* gene in the SNH 512, 256, and 128 μg/mL groups was down-regulated by 2.85-fold, 2.28-fold, and 1.37-fold, respectively, and the *EAP1* gene in the SNH 512, 256, and 128 μg/mL groups was down-regulated by 2.24-fold, 1.54-fold, and 1.18-fold, respectively. In addition, down-regulated the expression levels of the Ras1-cAMP-Efg1 pathway- related genes, *RAS1, EFG1, TEC1*, and *HWP1* (Figures 6C,D). Specifically, the *RAS1* gene in the SNH 512, 256, and 128 μg/mL groups was down-regulated by 5.36-fold, 2.64-fold, and 1.71-fold, respectively, and the *EFG1* gene in the SNH 512, 256, and 128 μg/mL groups was down-regulated by 1.83-fold, 1.38-fold, and 1.24-fold, respectively. The *TEC1* gene in the SNH 512, 256, and 128 μg/mL groups was down-regulated by 3.35-fold, 1.90-fold, and 1.52-fold, respectively, and the *HWP1* gene in the SNH 512, 256, and 128 μg/mL groups was down-regulated by 3.55-fold, 1.72-fold, and 1.39-fold, respectively. The *YWP1* and *RHD1* genes play a negative regulatory role in the adhesion of yeast-like cells, and the adhesion of biofilms formed by *YWP1*- or *RHD1*-deficient strains is enhanced (Figure 6E). The *YWP1* gene in the SNH 512, 256, and 128 μg/mL groups was up-regulated by 8.90-fold, 2.18-fold, and 1.93-fold, respectively, and the *RHD1* gene in the SNH 512, 256, and 128 μg/mL groups was up-regulated by 3.83-fold, 1.34-fold, and 1.10-fold, respectively.

To further explore the effects of SNH in the Ras1-cAMP-Efg1 pathway, we determined the production of cAMP in *C. albicans* treated with SNH at 4 h and 24 h after drug treatment. The results (Figure 6F) show that SNH can significantly reduce the production of cAMP both at 4 h and 24 h in a dose-dependent manner. The positive control, FLZ, also significantly inhibited the production of cAMP in *C. albicans*. Therefore, our results indicate that SNH can significantly reduce the production of cAMP which is the key messenger molecule of the Ras1-cAMP-Efg1 pathway in *C. albicans*. We speculated that the mechanism of SNH in *C. albicans* biofilm inhibition may be related to blocking the Ras1-cAMP-Efg1 signaling pathway.

### Determination of LD_50_ of *C. albicans* SC5314 Strains in *G. mellonella* Larvae

Inoculation of *G. mellonella* with *C. albicans* SC5314 strains resulted larval killing in a fungal concentration-dependent manner (Supplementary Figure S3). Based on the survival study, 8 × 10^4^ CFU/larvae was determined as the LD_50_ in *G. mellonella* larvae.

### SNH Effectively Treats *C. albican*s Infection *in vivo*

**FIGURE 7:**
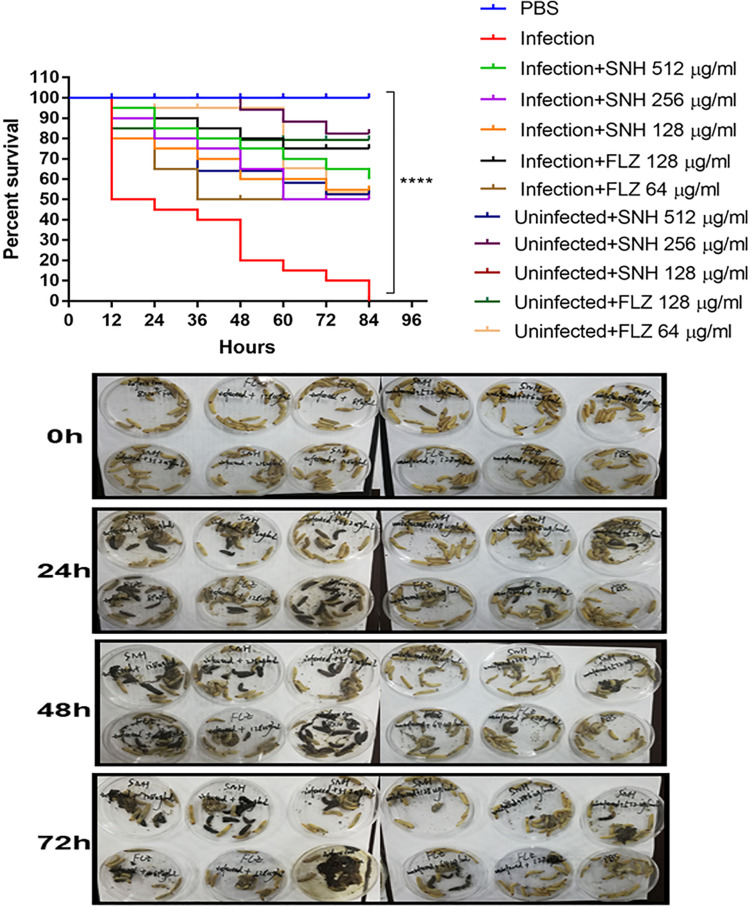
*In vivo* evaluation of antifungal efficacy of SNH against *C. albicans*. Survival of *G. mellonella* larvae infected with LD_50_ dose of *C. albicans* SC5314 strains (8 × 10^4^ CFU/larvae) and treated with SNH 1 h post-infection. *C. albicans* SC5314 induced infection (10 μL) was treated with SNH (10 μL), keeping respective controls (infected, infected with FLZ-treatment, PBS). Data expressed as the mean of three independent experiments. Survival curves were plotted using the Kaplan-Meier method and statistical analysis were performed using the log-rank test for multiple comparisons. ****p < 0.0001.

As shown in Figure 7, the survival rate in the PBS group was 100%. The survival rate in the infected group was only 50% after 12 h. In the next 72 h, larvae in the infected group continued to die, and the final survival rate of the infected group was 0% after 84 h. It can be seen that the 256 μg/mL and 128 μg/mL SNH control groups did not show obvious drug toxicity, and particularly in the uninfected + SNH 128 μg/mL group, no larvae died after 84 h. The survival rate in the uninfected + SNH 256 μg/mL group was 85% after 84 h, and the survival rate of the uninfected + SNH 512 μg/mL group was 55% after 84 h. These results indicate that SNH is slightly toxic at high concentrations, and SNH is not toxic at low concentrations. We further investigated the effects of SNH treatment at 1 h post-infection and found that the survival rate of the infection + SNH 512 μg/mL group was 60% after 60 h, the survival rate of the infection + SNH 256 μg/mL group was 50% after 84 h, and the survival rate of the infection + SNH 128 μg/mL group was 55% after 84 h. These results show that compared with the survival rate of the infected group, the survival rate of all SNH dose groups remained at least above 50%, and the difference was statistically significant (*p* < 0.0001). This indicates that SNH plays an obvious antifungal role in the body, thereby improving the survival rate of larvae infected by *C. albicans*.

## Discussion

The medical impact of *Candida albicans* is usually attributed to its ability to form biofilms, which are tightly packed cell communities that tightly adhere to a surface and are embedded in a protective polymer extracellular matrix (Simonetti et al., 2019). Fungal pathogens form biofilms that are highly resistant to antibacterial treatment. When yeast cells adhere to a solid surface in approximately 1–2 h, the adherent cells proliferate and divide early, followed by biofilm formation, forming a complex three-dimensional structure, which is bound together by hyphae and an outer polymer matrix. After 24 h of cultivation, a mature biofilm is formed, which is a network of yeast, hyphae, pseudohyphae and extracellular material (Ramage et al., 2005; Lohse et al., 2018). In the current study, we demonstrated that SNH is effective in inhibiting the adhesion and biofilm formation of *C. albicans*. Importantly, SNH not only suppresses adhesion but also inhibits the formation of a mature biofilm. Adhesion is the first stage of biofilm development (Douglas, 2003). The adhesion of *C. albicans* was significantly decreased after SNH treatment, indicating that SNH inhibits biofilm formation by suppressing adhesion (Li et al., 2017). Furthermore, to prevent the formation of biofilms, the transformation of yeast cells to hyphae should be prevented (Kavanaugh et al., 2014), and we found that SNH can significantly repress the yeast-to-hypha morphological transition, which is key to the biofilm maturation of *C. albicans*. The present results suggest that the anti-biofilm activity of SNH may be attributed to its anti-adhesion and anti-morphological transformation activities (Bachmann et al., 2002). Recently, Liu et al. (2020) revealed that sodium houttuyfonate, a derivative of SNH, in combination with berberine, palmatine, jatrorrhizine, and cinnamaldehyde can synergistically inhibit several *Candida* isolates, and induce cell wall remodeling of these isolates. In XTT reduction assays, we found that SNH had strong antifungal activity within 4 h of initial adhesion, which was significantly better than SNH antifungal activity during the 24-h biofilm formation phase. Through inverted microscope observations, we found that SNH can effectively inhibit *C. albicans* adhesion during the initial adhesion phase, and SNH can inhibit large thick biofilm formation during the biofilm phase, but there were still many single fungal cells or small biofilms observable through CLSM and SEM. To further examine the effect of SNH on *C. albicans* biofilms *in vitro*, we also selected four clinical strains of *C. albicans*, and found that SNH can still effectively inhibit biofilm formation through quantification and crystal violet staining. We speculated whether these SNH anti-biofilm effects are related to fungicidal activity. A previous study found that the antimicrobial peptide VLL-28 can inhibit yeast cell growth in the suspended state, prevent cell adhesion and eliminate established biofilms *in vitro*, and also found that VLL-28 can reduce the quantity of *Candida* biofilms, but with no fungicidal effects (Roscetto et al., 2018). Our research also found that SNH inhibits the adhesion and growth of yeast cells and is effective against biofilms. SNH can reduce *C. albicans* biofilms, but cannot effectively kill fungi. This result can be interpreted by the ability of SNH to affect the structure and stability of the biofilm matrix by interacting with one or more components, thereby inducing the decomposition of the biofilm without killing the cells.

Oral infections caused by *Candida* are usually biofilms, and *Candida* is the most common fungal pathogens in humans (Millsop and Fazel, 2016; Vipulanandan et al., 2018). Biofilm resistance is an important factor in human disease. *Candida* biofilms represent an important virulence factor, and their reduction is particularly important in combating infection (Stringaro et al., 2018). Among the phenotypic changes observed in cells that form part of biofilms, the most clinically relevant feature is their increased resistance to antifungal therapy (Mukherjee and Chandra, 2004). Compared with plankton, biofilm cells can display an up to 1000-fold increased resistance (Rossoni et al., 2019). Before conducting clinical trials, *in vivo* studies should be conducted to assess the potential antifungal effect of SNH in *C. albicans*. In determining the potential toxicity of fungal pathogens, mature *Galleria mellonella* larvae have proven to be a promising substitute for mammals (Glavis-Bloom et al., 2012; Arvanitis et al., 2013). The current study is the first to investigate the anti-*C. albicans* properties of SNH using *G. mellonella*. The results show that SNH has a therapeutic effect on *C. albicans* infection *in vivo*, and this is maintained after 24 h of mature biofilm formation. In the following 60 h, compared with the infection group where larvae continued to die until the survival rate was 0%, the SNH groups maintained a survival rate of more than 50%. The present study shows that SNH has an antifungal biofilm effect both *in vitro* and *in vivo*.

Because of the increased abuse of traditional antifungal drugs and antibiotics, the resistance of *C. albicans*, especially to FLZ, is increasing. Antifungal treatment faces serious hurdles, and there is an urgent need to find new antifungal drugs. To date, most of the reported chemicals that claim to have potential antifungal functions have a high MIC. However, these antifungal agents usually have a strong resistance reversal potential in FLZ-resistant *C. albicans* (Letscher-Bru et al., 2013; Padmavathi et al., 2015). Therefore, it may be necessary to find a new drug that can increase the antifungal activity of FLZ as another method to expand the antifungal library (Guo et al., 2008). Studies have found that SH and FLZ have a synergistic effect in FLZ-resistant *C. albicans*, and SH has strong potential to enhance FLZ treatment (Shao et al., 2017). Since higher MIC values may hinder the future development of pharmaceutical products, the combination of different forms of Chinese medicine monomers and traditional antifungal drugs is conducive to realizing the effectiveness of anti-fungal pathogens. Liu et al. reported a good antifungal effect through the combination of SH and various traditional Chinese medicine monomers (Liu et al., 2020). Here, we chose SNH and four antifungals and observed changes in the MIC and biofilm formation, and found that SNH combined with four antifungals has a significant antifungal biofilm effect. It is suggested that SH or SNH could have significant clinical potential in combination with different antifungals, in terms of reducing MIC, and producing significant anti-biofilm effects, providing treatment options for the clinical treatment of fungal infections.

With the development of genomics and the advent of whole genome sequencing platforms, metabolic pathways and related genes involved in fungal pathogenesis have been revealed (Chong et al., 2018). The current study found that the anti-adhesive effects of SNH are more significant than its anti-biofilm effects, and to verify that SNH may inhibit biofilm formation by inhibiting initial adhesion, we sequenced the transcriptome of *C. albicans* at an initial adhesion phase in 256 μg/mL (1 MIC_80_) SNH treatment and control groups through a high-throughput RNA sequencing method. Compared with the genomic data, the transcriptomic analysis provided more significant information about the relevant genes under different conditions (Grumaz et al., 2013; Giosa et al., 2017). To clarify the anti-adhesion and anti-biofilm mechanism of SNH, further PCR results were combined with GO enrichment analysis of the transcriptome sequencing results. Compared with the control group, the expression of 611 genes, including 361 down-regulated and 250 up-regulated genes, was significantly altered in the SNH group. Among these, several important adhesion- and biofilm-related genes were down-regulated after SNH treatment such as *RAS1, ALS3*, and *HWP1*, which are components of the Ras1-cAMP-Efg1 pathway. The down-regulation of these genes may contribute to the anti-adhesive and anti-biofilm activity of SNH. We found that the expression of *ALS1, ALA1, ALS3, EAP1, RAS1, EFG1, HWP1*, and *TEC1* in the Ras1-cAMP-Efg1 pathway of *C. albicans* was significantly down-regulated after SNH treatment in a dose-dependent manner and the expression of *YWP1* and *RHD1* of *C. albicans* was up-regulated after SNH treatment in a dose-dependent manner, as assessed through qRT-PCR and semi-qRT-PCR assays.

**FIGURE 8:**
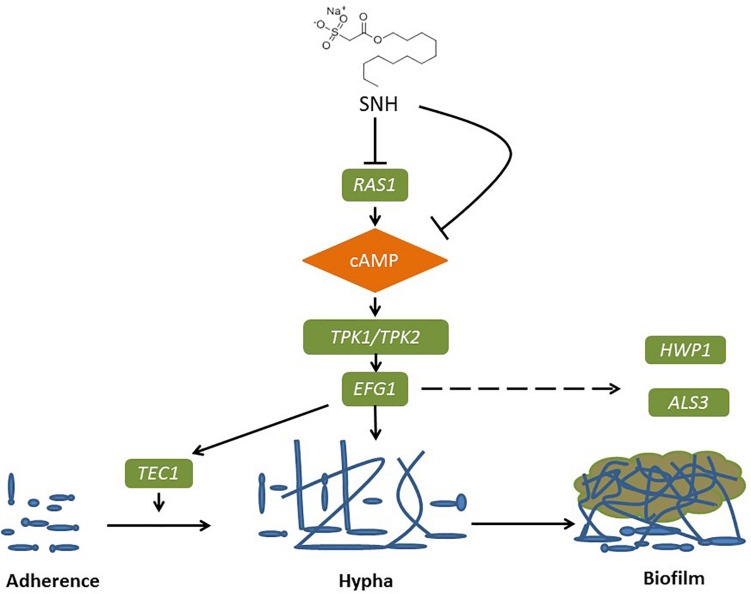
Possible mechanism of SNH inhibits the biofilm formation of *C. albicans* by repression of Ras1-cAMP-Efg1 pathway.

The Ras1-cAMP-Efg1 pathway is responsible for the adhesion, yeast-hyphal transition, biofilm formation and virulence of *C. albicans* (Davis-Hanna et al., 2008). Compounds of natural origin may represent a new strategy to prevent fungal adhesion and biofilm formation (De Vita et al., 2014). Certain natural products, such as magnolol and honokiol, can inhibit the adhesion, yeast-hyphal transition and biofilm formation of *C. albicans* through inactivation of the Ras1-cAMP-Efg1 pathway (Sun et al., 2015). More specifically, Ras plays a key role in controlling yeast/hyphal morphogenesis, cell adhesion and biofilm formation in *C. albicans* (Piispanen et al., 2011). Accordingly, *EFG1* is the central regulator of biofilm formation of *C. albicans* (Theberge et al., 2013). It is essential for Candidulin growth, and is considered to be essential for the interaction between *C. albicans* and human host cells (Stoldt et al., 1997). The *TEC1* gene, encoding transcription factors, positively regulates the expression of hypha-specific genes such as *HWP1* and *ALS3* (Panariello et al., 2017). The *ALS* gene family plays an essential role in the adherence stage of *C. albicans*, and contributes to the invasion of cells and subsequently host cell damage (Sundstrom, 2002; Liu and Filler, 2011). *ALA1* and *ALS1* encode proteins that increase adherence, and produce germ tubes that exhibit thigmotropic behavior and seek solid surfaces, and grow within host tissues (Hoyer et al., 1995; Sundstrom, 2002). *HWP1* codes for a cell surface glycoprotein targeted by mammalian transglutaminase that is required for cell adhesive interactions during biofilm formation (Ene and Bennett, 2009). The involvement of *HWP1* in *C. albicans* adhesion is supported by the *EAP1* gene which encodes a glucan-crosslinked cell wall protein (Li and Palecek, 2003). Similar to many other genes, *HWP1* and *EAP1* are downstream effectors of *EFG1* and *NRG1* as transcription factors (Li and Palecek, 2003; Remmele et al., 2015). Furthermore, expressed cell wall adhesions, including *ALS* and *HWP*, are crucial for *C. albicans* attachment to host tissue and for multispecies biofilm formation (Theberge et al., 2013). Notably, *ALS1, ALS3*, and *HWP1* play complementary roles during biofilm formation suggesting that they might interact to promote adhesion between adjacent cellular surfaces (Lo et al., 1997). Both *ALS3* and *HWP1* are developmentally regulated and exclusively expressed in *C. albicans* hyphae (Hoyer et al., 1998), which falls under the domain of the cAMP-protein kinase-A signaling pathway that regulates yeast-hypha morphogenesis via the transcription factor *EFG1* (Lo et al., 1997; Hoyer et al., 1998; Bastidas et al., 2009; Zhang et al., 2017). Overall, *HWP1* is a downstream component of the cAMP-dependent PKA pathway and is positively regulated by *EFG1* (Lin et al., 2018). The results of PCR and eukaryotic transcriptome sequencing show that SNH can inhibit the gene expression of *ALS1, ALA1, ALS3, EAP1, RAS1, EFG1, HWP1*, and *TEC1* in the Ras1-cAMP-Efg1 pathway of *C. albicans.* In addition, we determined the effects of SNH on the production of cAMP which is the key messenger molecule in the Ras1-cAMP-Efg1 pathway of *C. albicans*. Previous studies have found that the yeast-to-mycelium transition of *C. albicans* is positively regulated by the Ras1-cAMP-Efg1 and MAPK signaling pathways, while cAMP is an intermediate regulator of morphogenesis (Chang et al., 2012). The results showed that SNH can also effectively repress the production of cAMP at two different time points (4 and 24 h) throughout the *C. albicans* growth cycle. Combined with the transcriptome and qRT-PCR results, these data suggest that SNH inhibits the activity of the Ras-cAMP-Efg1 pathway and leads to an alteration to mechanisms underlying *C. albicans* adherence growth, yeast-hyphal transition and biofilm formation (Figure 8). In addition, a previous study found that *YWP1*-deficient blastoconidia exhibited increased adhesiveness and biofilm formation, suggesting that *YWP1* may promote the dispersal of yeast forms of *C. albicans* (Granger et al., 2005). We also evaluated the expression of the yeast-specific gene *RHD1*, and previous studies have shown that expression patterns of *Candida* species-related genes are significantly increased during hyphae-specific gene expression over the duration of biofilm formation. On the contrary, the expression of genes suppressing yeast-specific genes (*RHD1*) and filament formation was decreased (Park et al., 2014; Lee et al., 2017). Since *YWP1* and *RHD1* play a negative regulatory role in the adhesion of yeast-like cells, the adhesion of biofilms formed by *YWP1*- or *RHD1*-deficient strains is enhanced (Chandra et al., 2001; Znaidi et al., 2013). Therefore, we detected through PCR that the relative expression levels of *YWP1* and *RHD1* genes in the SNH treatment group were increased in the 24-h biofilm state compared with the control group.

It has been reported that FLZ can exert antifungal effects by regulating the Ras-cAMP-PKA signaling pathway (Liu et al., 2017; Sun et al., 2019). Coincidentally, our experimental results also found that FLZ can down-regulate the expression of genes related to the Ras1-cAMP-Efg1 signaling pathway and the secretion level of cAMP, which demonstrates that FLZ could exert antifungal effects by regulating the Ras1-cAMP-Efg1 signaling pathway. At present, there are no reports that SNH can exert antifungal effects through the Ras1-cAMP-Efg1 signaling pathway. However, we were surprised to find that SNH can down-regulate the expression of genes related to the Ras1-cAMP-Efg1 signaling pathway and the secretion level of cAMP. The mechanism of SNH against *C. albicans* may be related to the regulation of the Ras1-cAMP-Efg1 signaling pathway. In the future, studies are required to investigate the hyphae invasion/virulence of SNH-treated *C. albicans*, with relevant cell experiments to investigate possible mechanisms. In addition, by optimizing the chemical structure of SNH, its bioavailability was improved. Slow release drug delivery agents could also be used to prolong the release and action time of SNH.

## Conclusion

In summary, through *in vivo* and *in vitro* experiments, it was found that SNH has an obvious anti-*C. albicans* biofilm and infection effect. In addition, SNH combined with other antifungals can have synergistic efficacy in terms of anti-*C. albicans* biofilm effects. The results of eukaryotic transcriptome sequencing and PCR indicated that the anti-*C. albicans* biofilm mechanism of SNH may be closely related to the Ras1-cAMP-Efg1 pathway. Further gene knockout studies are required to verify that SNH regulates this pathway to inhibit adhesion, yeast hypha transition and biofilm formation. Our results reveal potential new applications for existing natural products of *Houttuynia cordata* which is an edible vegetable and traditional Chinese herb that has potential as a treatment to inhibit opportunistic fungal pathogens.

## Data Availability Statement

The datasets presented in this study can be found in online repositories. The names of the repository/repositories and accession number(s) can be found below: https://www.ncbi.nlm.nih.gov/sra/PRJNA544616, PRJNA544616.

## Author Contributions

DW, GY, and CW conceived and designed the study. JW and DW wrote the manuscript. JS and TW critically reviewed the manuscript and provided general advice. JW, YS, YZ, and LM performed the experiments and analyzed the data. All authors have read and agreed to the published version of the manuscript.

## Conflict of Interest

The authors declare that the research was conducted in the absence of any commercial or financial relationships that could be construed as a potential conflict of interest.

## Footnotes

1 https://github.com/jstjohn/SeqPrep

2 https://github.com/najoshi/sickle

3 http://tophat.cbcb.umd.edu/, version2.0.0

4 http://deweylab.biostat.wisc.edu/rsem/

5 http://www.bioconductor.org/packages/2.12/bioc/html/edgeR.html

6 https://github.com/tanghaibao/Goatools

7 http://kobas.cbi.pku.edu.cn/home.do

8 http://tophat.cbcb.umd.edu/, version2.0.0

9 http://rnaseq-mats.sourceforge.net/

10 www.i-sanger.com

## Supplementary Material

FIGURE S1 Volcano plot diagram of the extent of the gene differential expression between two groups.

FIGURE S2 Hierarchical cluster analysis of the 611 significantly expression changing genes between two groups.

FIGURE S3 Determination of LD_50_ of *C. albicans* SC5314 strains in *G. mellonella* larvae.

TABLE S1 Expression changes of several genes in Ras1-cAMP-Efg1 pathway determined by transcriptomic analysis.

TABLE S2 Complete list of genes with significantly expression changes determined by transcriptomic analysis.

## Abbreviations

AOD: average optical density
BBR: berberine chloride
cAMP: cyclic adenosine monophosphate
CAS: caspofungin
cDNA: complementary deoxyribonucleic acid
CFU: colony-forming unit
CLSM: confocal laser scanning microscopy
DEPC: diethypyrocarbonate
FIC: fractional inhibitory concentration
FICI: fractional inhibitory concentration index
FLZ: fluconazole
GO: gene ontology
ITZ: itraconazole
LD_50_: lethal dose 50%
MFC: minimum fungicidal concentrations
MIC_80_: minimum inhibitory concentrations of 80%
PBS: phosphate-buffered saline
PC: principal component
PCA: principle component analysis
PCR: polymerase chain reaction
qRT-PCR: quantitative reverse transcription polymerase chain reaction
RNA: ribonucleic acid
SEM: scanning electron microscopy
SNH: sodium new houttuyfonate
XTT: 2,3-Bis-(2-methoxy-4-nitro-5-sulfophenyl)-2H-tetrazolium-5-carboxanilide.
