# Supplementary material for: Sodium New Houttuyfonate Inhibits Candida albicans Biofilm Formation by Inhibiting the Ras1-cAMP-Efg1 Pathway Revealed by RNA-seq
Source: Front Microbiol. 2020 Aug 25;11:2075. doi: 10.3389/fmicb.2020.02075 (PMC7477049; doi:10.3389/fmicb.2020.02075)
Supplement: FIGURE S1 — Volcano plot diagram of the extent of the gene differential expression between two groups. [file Data_Sheet_1.docx]

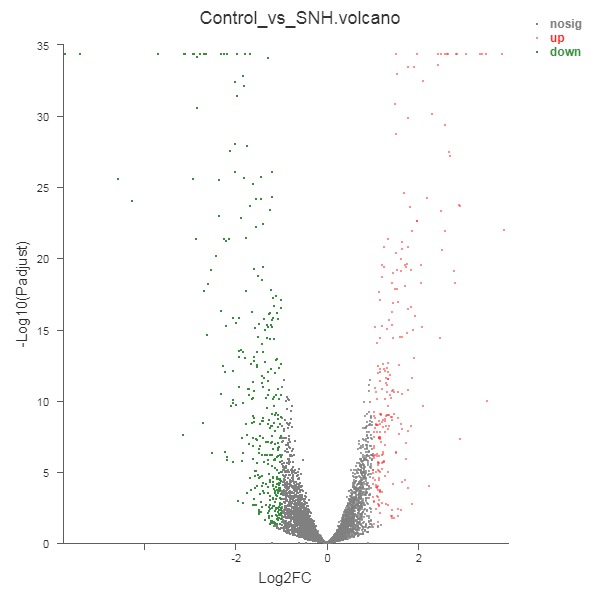


**Suppl Figure S1 ǀ** Volcano plot diagram of the extent of the gene differential expression between the two groups. The abscissa is the fold change value of the gene expression difference between the two samples, that is, the expression level of the treatment sample divided by the expression amount of the control sample, and the ordinate is the statistical test value of the difference in the gene expression amount change, that is, the p value. The higher the p value, the more significant the difference in expression, and the values of the horizontal and vertical coordinates are logarithmically processed. Each point in the figure represents a specific gene, the red dot indicates a significantly up-regulated gene, the blue dot indicates a significantly down-regulated gene, and the black dot is a non-significant differential gene. After mapping all the genes, it can be known that the gene on the left is the gene whose expression is down-regulated, and the one on the right is the gene whose expression is up-regulated, and the difference between the left and upper points is more significant.


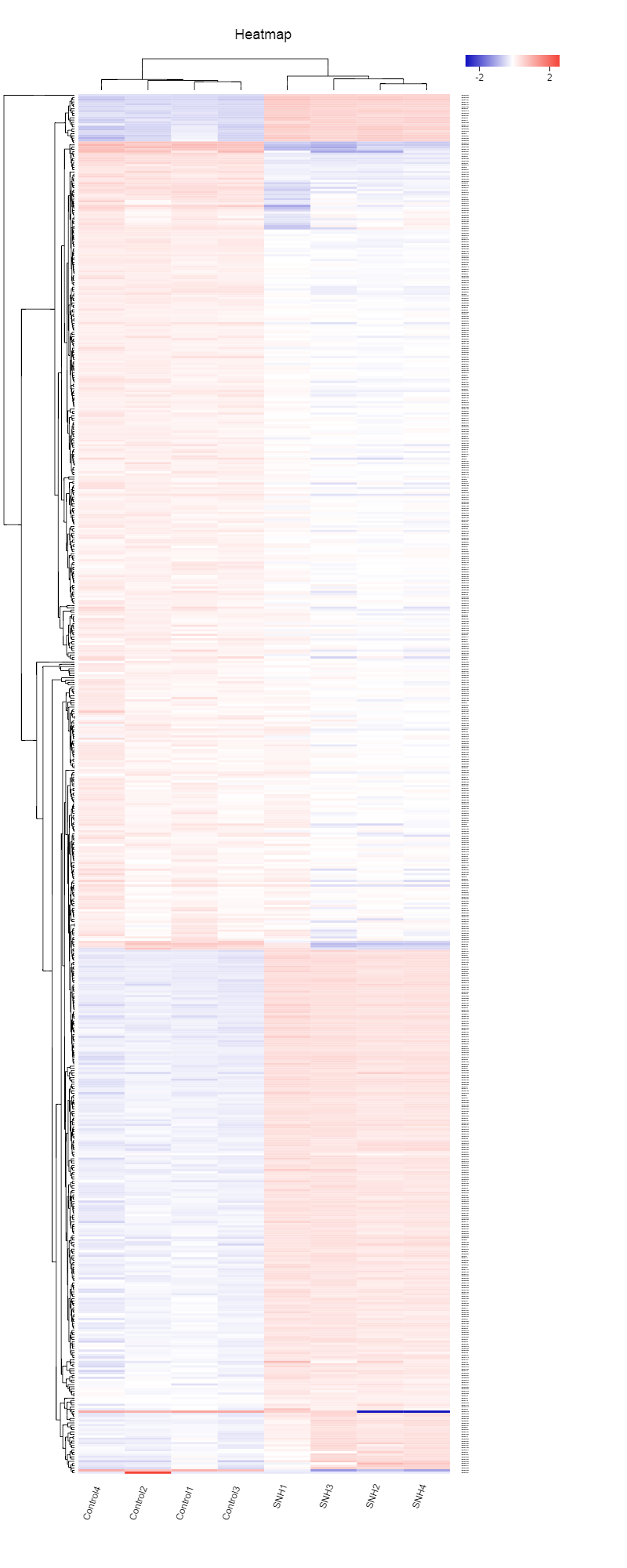


**Suppl Figure S2 ǀ** Hierarchical cluster analysis of the 611 significantly expression changing genes between two groups. Hierarchical cluster analysis was conducted for the expression of 611 genes of the samples [log_10_ (TPM+1)]. The color indicates relative fold changes (red = up-regulated, blue = down-regulated).


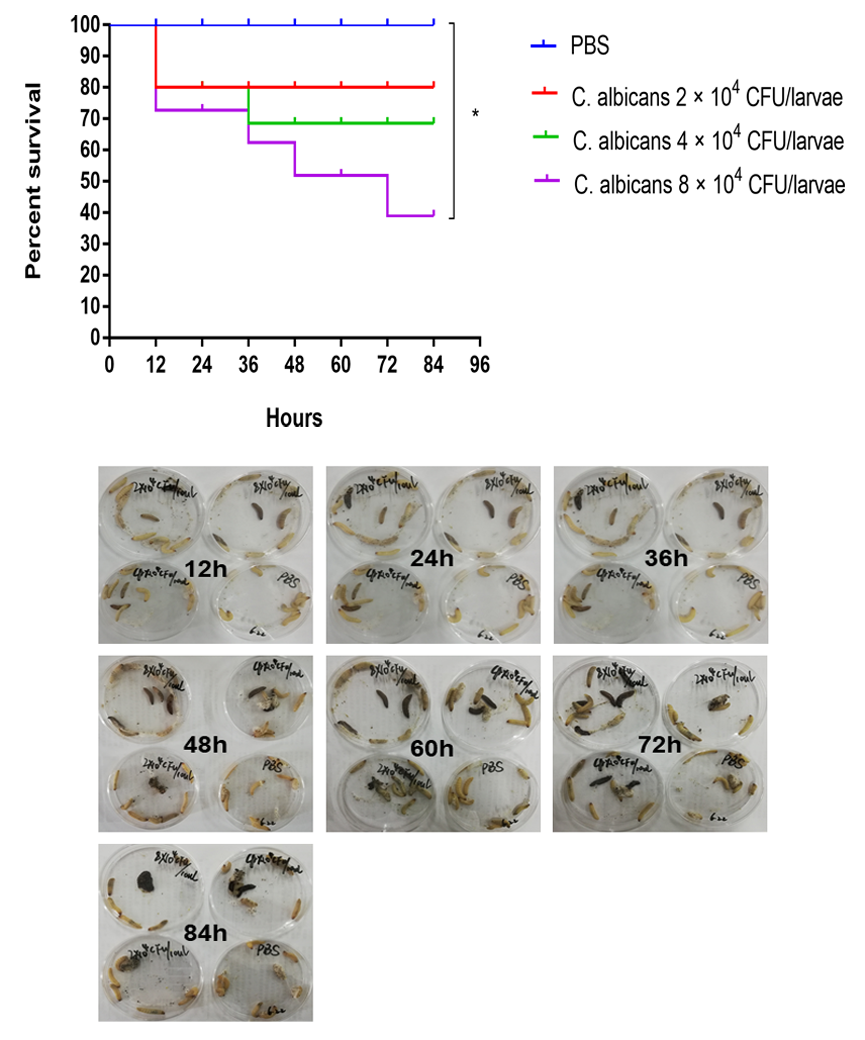


**Suppl Figure S3 ǀ** Determination of LD_50_ of *C. albicans* SC5314 strains in *G. mellonella* larvae. Data expressed as the mean of three independent experiments. Survival curves were plotted using the Kaplan-Meier method and statistical analysis were performed using the log-rank test for multiple comparisons. * *p* < 0.05.

**Suppl Table S1 ǀ** Expression changes of several genes in Ras1-cAMP-Efg1 pathway determined by transcriptomic analysis

| Gene ID | Gene Name | Fold Change (SNH/Control) | P value | Significant | Regulation |
| --- | --- | --- | --- | --- | --- |
| Gene2381 | *RAS1* | 0.353 | 1.1E-17 | Yes | Down |
| Gene5910 | *ALS3* | 0.159 | 1.12E-51 | Yes | Down |
| Gene3543 | *HWP1* | 0.019 | 2.6E-112 | Yes | Down |

**Suppl Table S2 ǀ** Complete list of genes with significantly expression changes determined by transcriptomic analysis

| Gene ID | Gene Name | Fold Change (SNH/Control) | P value | Significant | Regulation |
| --- | --- | --- | --- | --- | --- |
| gene5475 | OPT4 | 0.024 | 2.1754E-118 | yes | down |
| gene4450 | FET34 | 0.076 | 3.3955E-118 | yes | down |
| gene3543 | HWP1 | 0.019 | 2.6252E-112 | yes | down |
| gene852 | CAALFM_C108900WA | 0.146 | 4.5679E-74 | yes | down |
| gene2584 | CAALFM_C301540WA | 0.116 | 2.80621E-60 | yes | down |
| gene2035 | CAALFM_C206570CA | 0.132 | 1.15373E-59 | yes | down |
| gene688 | CAALFM_C107160CA | 0.113 | 4.77755E-59 | yes | down |
| gene5910 | ALS3 | 0.159 | 1.11684E-51 | yes | down |
| gene4422 | PGA48 | 0.287 | 1.41475E-49 | yes | down |
| gene25 | CAALFM_C100310WA | 0.209 | 1.45962E-46 | yes | down |
| gene2459 | CAALFM_C300230CA | 0.076 | 2.79783E-46 | yes | down |
| gene5333 | ATO9 | 0.255 | 4.41451E-46 | yes | down |
| gene4627 | CAALFM_C602330WA | 0.218 | 4.23694E-45 | yes | down |
| gene1498 | HGT6 | 0.13 | 3.97957E-44 | yes | down |
| gene6170 | DEF1 | 0.201 | 5.12156E-43 | yes | down |
| gene1308 | ROB1 | 0.153 | 2.14653E-41 | yes | down |
| gene3818 | CAALFM_C406520WA | 0.311 | 5.29215E-39 | yes | down |
| gene2458 | HGT19 | 0.139 | 3.72444E-37 | yes | down |
| gene398 | HTA2 | 0.411 | 4.57515E-37 | yes | down |
| gene3218 | RBT5 | 0.277 | 9.15786E-36 | yes | down |
| gene2037 | CAALFM_C206600WA | 0.247 | 2.75345E-35 | yes | down |
| gene6260 | XYL2 | 0.283 | 5.05701E-35 | yes | down |
| gene3250 | PGA10 | 0.254 | 2.72455E-34 | yes | down |
| gene6073 | ITS1 | 0.139 | 1.90525E-33 | yes | down |
| gene2343 | CAALFM_C209820WA | 0.247 | 7.06957E-31 | yes | down |
| gene2762 | FCY2 | 0.296 | 1.0277E-30 | yes | down |
| gene6099 | CAALFM_CR09070CA | 0.231 | 2.40904E-30 | yes | down |
| gene6115 | CBP1 | 0.432 | 7.63325E-29 | yes | down |
| gene1467 | CAALFM_C200690WA | 0.247 | 7.85898E-29 | yes | down |
| gene4398 | PGA4 | 0.368 | 1.70137E-28 | yes | down |
| gene4486 | CAN3 | 0.285 | 2.12251E-28 | yes | down |
| gene439 | CAALFM_C104590WA | 0.131 | 2.42072E-28 | yes | down |
| gene1466 | SOD5 | 0.042 | 2.78607E-28 | yes | down |
| gene5273 | CAALFM_CR00310CA | 0.194 | 3.11342E-28 | yes | down |
| gene1535 | CLB2 | 0.326 | 6.17383E-28 | yes | down |
| gene399 | CAALFM_C104180WA | 0.434 | 5.0523E-27 | yes | down |
| gene5118 | CAALFM_C703030WA | 0.369 | 6.99204E-27 | yes | down |
| gene4932 | CAALFM_C700910CA | 0.342 | 7.22478E-27 | yes | down |
| gene4773 | IHD1 | 0.052 | 1.06621E-26 | yes | down |
| gene6211 | PTP3 | 0.311 | 2.20115E-26 | yes | down |
| gene406 | HHT21 | 0.422 | 5.13746E-26 | yes | down |
| gene5968 | CAALFM_CR07680CA | 0.195 | 1.35476E-25 | yes | down |
| gene2689 | GLX3 | 0.27 | 1.85366E-25 | yes | down |
| gene3824 | CBF1 | 0.376 | 5.14919E-25 | yes | down |
| gene4718 | AYR2 | 0.341 | 8.38712E-25 | yes | down |
| gene2457 | CAALFM_C300210CA | 0.293 | 5.0646E-24 | yes | down |
| gene3158 | CAALFM_C307470WA | 0.225 | 5.67622E-24 | yes | down |
| gene4872 | HGT12 | 0.137 | 6.37943E-24 | yes | down |
| gene6079 | NIT2 | 0.209 | 6.55461E-24 | yes | down |
| gene4352 | MAL31 | 0.215 | 8.70256E-24 | yes | down |
| gene82 | CAALFM_C100880WA | 0.184 | 9.99899E-23 | yes | down |
| gene5885 | HHT2 | 0.379 | 6.58285E-22 | yes | down |
| gene4551 | CAALFM_C601530CA | 0.328 | 8.97868E-22 | yes | down |
| gene4843 | NAG4 | 0.172 | 1.10679E-21 | yes | down |
| gene4886 | CAALFM_C700420CA | 0.351 | 3.24305E-21 | yes | down |
| gene2146 | CAALFM_C207720CA | 0.373 | 6.53072E-21 | yes | down |
| gene684 | GAP4 | 0.165 | 1.16985E-20 | yes | down |
| gene405 | HHF1 | 0.429 | 3.18537E-20 | yes | down |
| gene6075 | ITS2 | 0.154 | 4.04645E-20 | yes | down |
| gene565 | PRN4 | 0.292 | 4.15214E-20 | yes | down |
| gene3368 | NHP6A | 0.462 | 8.61492E-20 | yes | down |
| gene662 | MMD1 | 0.438 | 1.2595E-19 | yes | down |
| gene5293 | ADE1 | 0.496 | 1.63688E-19 | yes | down |
| gene3953 | ERG11 | 0.445 | 4.93125E-19 | yes | down |
| gene3352 | ERG251 | 0.493 | 7.15366E-19 | yes | down |
| gene6076 | RDN25 | 0.2 | 1.084E-18 | yes | down |
| gene1126 | CAALFM_C111680CA | 0.322 | 1.33034E-18 | yes | down |
| gene1715 | GPM1 | 0.471 | 1.54351E-18 | yes | down |
| gene6065 | ARF1 | 0.419 | 1.68334E-18 | yes | down |
| gene3217 | PGA7 | 0.415 | 1.93006E-18 | yes | down |
| gene5752 | PST3 | 0.439 | 3.53255E-18 | yes | down |
| gene6104 | CAALFM_CR09140CA | 0.261 | 3.59492E-18 | yes | down |
| gene5452 | OPT2 | 0.239 | 3.69162E-18 | yes | down |
| gene4166 | GRE3 | 0.477 | 4.18226E-18 | yes | down |
| gene1790 | DCK1 | 0.385 | 4.75877E-18 | yes | down |
| gene1524 | MNN24 | 0.432 | 5.25359E-18 | yes | down |
| gene4842 | NAG3 | 0.25 | 8.26657E-18 | yes | down |
| gene2381 | RAS1 | 0.353 | 1.10174E-17 | yes | down |
| gene3446 | CAALFM_C402510WA | 0.401 | 1.11535E-17 | yes | down |
| gene5132 | HHO1 | 0.425 | 1.24737E-17 | yes | down |
| gene5357 | CAALFM_CR01220WA | 0.217 | 1.45116E-17 | yes | down |
| gene5330 | CAALFM_CR00910WA | 0.406 | 1.65961E-17 | yes | down |
| gene3823 | PDC11 | 0.435 | 1.65848E-17 | yes | down |
| gene2961 | CAALFM_C305450CA | 0.336 | 2.07382E-17 | yes | down |
| gene1327 | CAALFM_C113810WA | 0.389 | 2.91695E-17 | yes | down |
| gene3747 | CFL2 | 0.162 | 7.15599E-17 | yes | down |
| gene1630 | CAALFM_C202390WA | 0.352 | 8.83853E-17 | yes | down |
| gene3242 | HOF1 | 0.402 | 1.16704E-16 | yes | down |
| gene2816 | HTA1 | 0.448 | 1.38181E-16 | yes | down |
| gene457 | ERG3 | 0.422 | 1.44108E-16 | yes | down |
| gene5202 | CLB4 | 0.372 | 2.85691E-16 | yes | down |
| gene207 | CAALFM_C102210WA | 0.27 | 8.64003E-16 | yes | down |
| gene1357 | RBE1 | 0.264 | 9.45335E-16 | yes | down |
| gene3157 | CAALFM_C307460WA | 0.379 | 1.04292E-15 | yes | down |
| gene3400 | RFX2 | 0.343 | 1.11889E-15 | yes | down |
| gene602 | UME6 | 0.278 | 1.16329E-15 | yes | down |
| gene5001 | CAALFM_C701680CA | 0.261 | 2.68549E-15 | yes | down |
| gene3993 | CAALFM_C501070CA | 0.332 | 2.7589E-15 | yes | down |
| gene6190 | CHT3 | 0.288 | 3.01171E-15 | yes | down |
| gene3183 | WOR4 | 0.465 | 3.38488E-15 | yes | down |
| gene813 | ENO1 | 0.459 | 4.62351E-15 | yes | down |
| gene3974 | GIT3 | 0.328 | 4.75E-15 | yes | down |
| gene4885 | CAALFM_C700410CA | 0.387 | 5.81448E-15 | yes | down |
| gene1000 | FGR41 | 0.314 | 8.12148E-15 | yes | down |
| gene2196 | CAALFM_C208240WA | 0.496 | 8.34095E-15 | yes | down |
| gene4561 | FMP27 | 0.345 | 1.05116E-14 | yes | down |
| gene4594 | PLB1 | 0.206 | 1.11138E-14 | yes | down |
| gene4487 | GPX2 | 0.346 | 1.39282E-14 | yes | down |
| gene1116 | CAALFM_C111580WA | 0.48 | 1.58813E-14 | yes | down |
| gene2521 | CSE4 | 0.425 | 2.04322E-14 | yes | down |
| gene1878 | ABP2 | 0.241 | 2.64165E-14 | yes | down |
| gene5591 | SMC1 | 0.405 | 3.47298E-14 | yes | down |
| gene386 | MFALPHA | 0.211 | 3.47503E-14 | yes | down |
| gene3523 | VTC4 | 0.454 | 3.7562E-14 | yes | down |
| gene3653 | CAALFM_C404720WA | 0.454 | 4.19164E-14 | yes | down |
| gene1030 | CAALFM_C110710CA | 0.37 | 6.23127E-14 | yes | down |
| gene5490 | RFG1 | 0.382 | 8.58232E-14 | yes | down |
| gene529 | CAALFM_C105520WA | 0.405 | 1.42492E-13 | yes | down |
| gene2418 | CAALFM_C210650WA | 0.325 | 1.84478E-13 | yes | down |
| gene2942 | CAALFM_C305250CA | 0.371 | 2.06449E-13 | yes | down |
| gene2188 | PHM7 | 0.379 | 4.17857E-13 | yes | down |
| gene4240 | CAALFM_C503770CA | 0.263 | 4.27327E-13 | yes | down |
| gene2409 | ZSF1 | 0.476 | 5.66446E-13 | yes | down |
| gene676 | CAALFM_C107040CA | 0.299 | 6.07474E-13 | yes | down |
| gene150 | CAALFM_C101620CA | 0.306 | 6.3126E-13 | yes | down |
| gene5131 | IFA21 | 0.415 | 6.84262E-13 | yes | down |
| gene211 | CWH8 | 0.332 | 7.88072E-13 | yes | down |
| gene22 | CAALFM_C100270WA | 0.2 | 1.28945E-12 | yes | down |
| gene3167 | TCC1 | 0.496 | 1.64475E-12 | yes | down |
| gene6083 | ASR2 | 0.308 | 1.71545E-12 | yes | down |
| gene2232 | PST2 | 0.463 | 1.72497E-12 | yes | down |
| gene1078 | ARL3 | 0.406 | 1.76084E-12 | yes | down |
| gene3589 | RHD3 | 0.446 | 2.40651E-12 | yes | down |
| gene1292 | HYR1 | 0.327 | 2.71255E-12 | yes | down |
| gene1125 | CAALFM_C111670WA | 0.398 | 2.90012E-12 | yes | down |
| gene4179 | CAALFM_C503080CA | 0.32 | 3.07313E-12 | yes | down |
| gene89 | CDC5 | 0.482 | 3.06327E-12 | yes | down |
| gene244 | SNO1 | 0.441 | 3.45379E-12 | yes | down |
| gene6072 | RDN18 | 0.239 | 4.22851E-12 | yes | down |
| gene5932 | FRE7 | 0.238 | 6.19132E-12 | yes | down |
| gene5154 | CAALFM_C703410CA | 0.279 | 6.87116E-12 | yes | down |
| gene3447 | CAALFM_C402520CA | 0.249 | 8.52691E-12 | yes | down |
| gene449 | CAALFM_C104690CA | 0.448 | 1.06693E-11 | yes | down |
| gene1926 | CDC19 | 0.449 | 1.08885E-11 | yes | down |
| gene1786 | HSP21 | 0.231 | 1.16462E-11 | yes | down |
| gene2527 | ATO2 | 0.404 | 1.42455E-11 | yes | down |
| gene5955 | ECM22 | 0.461 | 3.6907E-11 | yes | down |
| gene5884 | HHF22 | 0.431 | 4.25741E-11 | yes | down |
| gene4933 | CAALFM_C700920CA | 0.471 | 4.79781E-11 | yes | down |
| gene2754 | HTA3 | 0.453 | 5.02929E-11 | yes | down |
| gene2036 | GIT1 | 0.327 | 6.1409E-11 | yes | down |
| gene5367 | CAALFM_CR01320CA | 0.494 | 7.10633E-11 | yes | down |
| gene1124 | GAD1 | 0.378 | 8.18942E-11 | yes | down |
| gene4543 | CAALFM_C601450CA | 0.328 | 8.65092E-11 | yes | down |
| gene1265 | TYE7 | 0.473 | 1.07623E-10 | yes | down |
| gene1589 | SRD1 | 0.412 | 1.11447E-10 | yes | down |
| gene763 | CAALFM_C107980CA | 0.379 | 1.40964E-10 | yes | down |
| gene5861 | CAALFM_CR06570CA | 0.371 | 1.50232E-10 | yes | down |
| gene4036 | CAALFM_C501530CA | 0.33 | 1.63903E-10 | yes | down |
| gene254 | CAALFM_C102700CA | 0.45 | 1.90148E-10 | yes | down |
| gene5500 | PGA34 | 0.152 | 1.98661E-10 | yes | down |
| gene6074 | RDN58 | 0.318 | 2.20307E-10 | yes | down |
| gene2131 | RNR22 | 0.488 | 2.3181E-10 | yes | down |
| gene2391 | KIP2 | 0.292 | 2.32602E-10 | yes | down |
| gene4256 | CAALFM_C503930CA | 0.363 | 2.39094E-10 | yes | down |
| gene4088 | CAALFM_C502110WA | 0.407 | 2.43113E-10 | yes | down |
| gene4087 | HSP12 | 0.407 | 2.50485E-10 | yes | down |
| gene5920 | CAALFM_CR07170WA | 0.425 | 3.37138E-10 | yes | down |
| gene531 | CAALFM_C105540CA | 0.432 | 3.4434E-10 | yes | down |
| gene299 | CAALFM_C103150CA | 0.476 | 4.25787E-10 | yes | down |
| gene3492 | CAALFM_C403000CA | 0.42 | 4.82582E-10 | yes | down |
| gene3654 | BAS1 | 0.455 | 8.59813E-10 | yes | down |
| gene3533 | ECE1 | 0.112 | 1.55846E-09 | yes | down |
| gene5474 | CAALFM_CR02470WA | 0.296 | 1.71756E-09 | yes | down |
| gene1313 | OSM2 | 0.495 | 1.79459E-09 | yes | down |
| gene1215 | SPA2 | 0.433 | 1.8428E-09 | yes | down |
| gene5750 | CAALFM_CR05370WA | 0.364 | 1.99818E-09 | yes | down |
| gene5571 | CAALFM_CR03480WA | 0.325 | 2.77034E-09 | yes | down |
| gene2179 | RPR1 | 0.378 | 3.00962E-09 | yes | down |
| gene4021 | CFL5 | 0.276 | 3.0206E-09 | yes | down |
| gene3458 | HFL2 | 0.492 | 3.02744E-09 | yes | down |
| gene5956 | CAALFM_CR07540CA | 0.342 | 3.64514E-09 | yes | down |
| gene5738 | CAALFM_CR05210WA | 0.344 | 3.73334E-09 | yes | down |
| gene5505 | CAALFM_CR02800CA | 0.397 | 5.41518E-09 | yes | down |
| gene1156 | CAALFM_C111990WA | 0.431 | 6.12543E-09 | yes | down |
| gene1158 | KIP4 | 0.406 | 6.15852E-09 | yes | down |
| gene3963 | FOL1 | 0.476 | 6.673E-09 | yes | down |
| gene5612 | WOR3 | 0.426 | 6.70092E-09 | yes | down |
| gene5144 | CAALFM_C703310WA | 0.304 | 7.49706E-09 | yes | down |
| gene3984 | TRY3 | 0.457 | 7.81865E-09 | yes | down |
| gene4281 | MRV2 | 0.323 | 1.08236E-08 | yes | down |
| gene185 | HGT1 | 0.36 | 1.10311E-08 | yes | down |
| gene3556 | CAALFM_C403700WA | 0.48 | 1.39315E-08 | yes | down |
| gene3899 | CAALFM_C500100CA | 0.479 | 1.71579E-08 | yes | down |
| gene4974 | CAALFM_C701400CA | 0.357 | 1.84514E-08 | yes | down |
| gene6223 | CAALFM_CR10460WA | 0.442 | 2.07943E-08 | yes | down |
| gene4976 | GIR2 | 0.477 | 2.28941E-08 | yes | down |
| gene2144 | CAALFM_C207700CA | 0.211 | 3.22462E-08 | yes | down |
| gene6210 | PGA11 | 0.277 | 3.55172E-08 | yes | down |
| gene5272 | CDA2 | 0.354 | 3.8095E-08 | yes | down |
| gene5568 | HXT5 | 0.174 | 3.97157E-08 | yes | down |
| gene204 | GAL7 | 0.399 | 5.38742E-08 | yes | down |
| gene990 | GCA1 | 0.492 | 5.5574E-08 | yes | down |
| gene2567 | IRO1 | 0.403 | 5.58623E-08 | yes | down |
| gene3954 | HMS1 | 0.433 | 5.6694E-08 | yes | down |
| gene6167 | CAALFM_CR09840CA | 0.418 | 5.83154E-08 | yes | down |
| gene4298 | PGA37 | 0.37 | 5.82596E-08 | yes | down |
| gene1196 | PKH2 | 0.392 | 5.84912E-08 | yes | down |
| gene1690 | RNR21 | 0.475 | 6.9803E-08 | yes | down |
| gene5644 | QDR1 | 0.495 | 7.30457E-08 | yes | down |
| gene553 | PGA26 | 0.22 | 7.39939E-08 | yes | down |
| gene2217 | DSE1 | 0.468 | 8.05668E-08 | yes | down |
| gene151 | CAALFM_C101630WA | 0.326 | 1.16495E-07 | yes | down |
| gene607 | CAALFM_C106350WA | 0.455 | 1.16684E-07 | yes | down |
| gene4985 | CAALFM_C701510WA | 0.454 | 1.23168E-07 | yes | down |
| gene3872 | CAALFM_C407080CA | 0.407 | 1.32411E-07 | yes | down |
| gene6143 | CAALFM_CR09590WA | 0.447 | 1.33631E-07 | yes | down |
| gene4855 | SOD3 | 0.22 | 1.44053E-07 | yes | down |
| gene3536 | CAALFM_C403500CA | 0.274 | 1.595E-07 | yes | down |
| gene1293 | KNS1 | 0.387 | 1.82908E-07 | yes | down |
| gene4485 | SUN41 | 0.486 | 1.8956E-07 | yes | down |
| gene6077 | TAR1 | 0.238 | 2.00482E-07 | yes | down |
| gene562 | POL32 | 0.345 | 2.30966E-07 | yes | down |
| gene1898 | WH11 | 0.407 | 2.85871E-07 | yes | down |
| gene4983 | CAALFM_C701490WA | 0.432 | 4.05434E-07 | yes | down |
| gene1241 | CAALFM_C112880CA | 0.453 | 5.20859E-07 | yes | down |
| gene4042 | CAALFM_C501590WA | 0.466 | 8.00371E-07 | yes | down |
| gene5854 | CAALFM_CR06500CA | 0.341 | 8.51492E-07 | yes | down |
| gene2697 | SMC3 | 0.332 | 9.34627E-07 | yes | down |
| gene1764 | ASR3 | 0.498 | 1.07771E-06 | yes | down |
| gene4283 | MRV4 | 0.498 | 1.18768E-06 | yes | down |
| gene4887 | CAALFM_C700430WA | 0.482 | 1.22712E-06 | yes | down |
| gene2736 | USO5 | 0.414 | 1.56949E-06 | yes | down |
| gene1256 | CAALFM_C113040CA | 0.351 | 1.82466E-06 | yes | down |
| gene1213 | CHR1 | 0.375 | 2.15628E-06 | yes | down |
| gene5338 | CAALFM_CR01020CA | 0.336 | 2.36679E-06 | yes | down |
| gene2945 | CAALFM_C305290CA | 0.363 | 2.54753E-06 | yes | down |
| gene5788 | CAALFM_CR05760CA | 0.47 | 2.68254E-06 | yes | down |
| gene3318 | ASK1 | 0.466 | 2.74329E-06 | yes | down |
| gene3499 | CAALFM_C403080WA | 0.44 | 2.92422E-06 | yes | down |
| gene6134 | CAALFM_CR09490WA | 0.309 | 3.07094E-06 | yes | down |
| gene6105 | CAALFM_CR09150WA | 0.356 | 3.32339E-06 | yes | down |
| gene78 | NRM1 | 0.439 | 3.38944E-06 | yes | down |
| gene2592 | CAALFM_C301620WA | 0.469 | 3.83344E-06 | yes | down |
| gene663 | CAALFM_C106910CA | 0.49 | 3.83754E-06 | yes | down |
| gene3330 | CAALFM_C401280CA | 0.473 | 3.90731E-06 | yes | down |
| gene1862 | CAALFM_C204780WA | 0.49 | 4.60711E-06 | yes | down |
| gene1532 | PLB4.5 | 0.486 | 4.80112E-06 | yes | down |
| gene6175 | CAALFM_CR09930WA | 0.422 | 4.88501E-06 | yes | down |
| gene5542 | CAALFM_CR03190CA | 0.422 | 4.89118E-06 | yes | down |
| gene3615 | FRE10 | 0.5 | 5.22079E-06 | yes | down |
| gene1200 | CAALFM_C112470WA | 0.441 | 6.8416E-06 | yes | down |
| gene5734 | FDH1 | 0.422 | 7.06243E-06 | yes | down |
| gene3439 | PGA38 | 0.493 | 7.72931E-06 | yes | down |
| gene162 | CTN1 | 0.375 | 8.16398E-06 | yes | down |
| gene158 | ARP8 | 0.481 | 8.5819E-06 | yes | down |
| gene1846 | CAALFM_C204610CA | 0.47 | 9.46138E-06 | yes | down |
| gene3310 | HGT17 | 0.399 | 9.4579E-06 | yes | down |
| gene1649 | ZRT2 | 0.439 | 1.12614E-05 | yes | down |
| gene6152 | RTA4 | 0.438 | 1.14737E-05 | yes | down |
| gene2624 | CAALFM_C301950CA | 0.482 | 1.19814E-05 | yes | down |
| gene4980 | CAALFM_C701460CA | 0.345 | 1.33832E-05 | yes | down |
| gene3929 | CAALFM_C500400CA | 0.37 | 1.33935E-05 | yes | down |
| gene1586 | AMN1 | 0.442 | 1.37439E-05 | yes | down |
| gene3338 | ZCF27 | 0.438 | 1.47641E-05 | yes | down |
| gene1103 | GIN1 | 0.408 | 1.65083E-05 | yes | down |
| gene2947 | USO6 | 0.462 | 1.74392E-05 | yes | down |
| gene4113 | CAALFM_C502370CA | 0.487 | 1.99949E-05 | yes | down |
| gene2063 | LTE1 | 0.333 | 2.16373E-05 | yes | down |
| gene5506 | CIRT4B | 0.479 | 2.16656E-05 | yes | down |
| gene5855 | CAALFM_CR06510WA | 0.444 | 2.47491E-05 | yes | down |
| gene4538 | CAALFM_C601400WA | 0.472 | 2.63847E-05 | yes | down |
| gene2652 | YCG1 | 0.469 | 2.67537E-05 | yes | down |
| gene859 | CAALFM_C108970WA | 0.315 | 2.85047E-05 | yes | down |
| gene3168 | CAALFM_C307570CA | 0.49 | 3.09629E-05 | yes | down |
| gene4533 | CAALFM_C601350WA | 0.376 | 3.1639E-05 | yes | down |
| gene1079 | CAALFM_C111200WA | 0.452 | 3.30513E-05 | yes | down |
| gene613 | CAALFM_C106410WA | 0.479 | 3.56203E-05 | yes | down |
| gene5824 | RPL7 | 0.472 | 4.16129E-05 | yes | down |
| gene4105 | PDE1 | 0.475 | 4.72129E-05 | yes | down |
| gene2991 | SAM50 | 0.452 | 5.3217E-05 | yes | down |
| gene1341 | CAALFM_C113950CA | 0.381 | 5.33389E-05 | yes | down |
| gene5331 | CCE1 | 0.295 | 5.33721E-05 | yes | down |
| gene2955 | GPI1 | 0.407 | 5.37138E-05 | yes | down |
| gene4798 | SPB1 | 0.359 | 5.6951E-05 | yes | down |
| gene2699 | CAALFM_C302720WA | 0.499 | 5.88208E-05 | yes | down |
| gene4253 | PGA56 | 0.457 | 7.0641E-05 | yes | down |
| gene2543 | CAALFM_C301100WA | 0.465 | 8.14629E-05 | yes | down |
| gene5199 | OGG1 | 0.418 | 8.53788E-05 | yes | down |
| gene3706 | USO1 | 0.471 | 9.12679E-05 | yes | down |
| gene3319 | CRD2 | 0.493 | 0.000127024 | yes | down |
| gene3455 | CAALFM_C402600CA | 0.438 | 0.000129012 | yes | down |
| gene5746 | CAALFM_CR05330WA | 0.329 | 0.000146717 | yes | down |
| gene2199 | EAF3 | 0.463 | 0.000149296 | yes | down |
| gene4521 | EBP1 | 0.498 | 0.000162344 | yes | down |
| gene4353 | CAALFM_C504940WA | 0.438 | 0.0001633 | yes | down |
| gene4610 | CAALFM_C602160WA | 0.421 | 0.000169806 | yes | down |
| gene194 | HSP31 | 0.26 | 0.000221089 | yes | down |
| gene5447 | PHO113 | 0.367 | 0.000270416 | yes | down |
| gene2934 | WOR2 | 0.456 | 0.000270644 | yes | down |
| gene3296 | CAALFM_C400910CA | 0.49 | 0.00028581 | yes | down |
| gene186 | HSP30 | 0.278 | 0.000317615 | yes | down |
| gene4621 | ALG11 | 0.494 | 0.000386746 | yes | down |
| gene24 | POL93 | 0.326 | 0.000412807 | yes | down |
| gene6082 | CAALFM_CR08880CA | 0.486 | 0.000412541 | yes | down |
| gene2145 | CAALFM_C207710WA | 0.333 | 0.000414323 | yes | down |
| gene4208 | CAALFM_C503430WA | 0.338 | 0.000433545 | yes | down |
| gene4534 | CAALFM_C601360WA | 0.423 | 0.00047694 | yes | down |
| gene2455 | CAALFM_C300190WA | 0.397 | 0.000482555 | yes | down |
| gene1842 | CAALFM_C204570WA | 0.448 | 0.000505286 | yes | down |
| gene5900 | CAALFM_CR06970CA | 0.437 | 0.000565101 | yes | down |
| gene2656 | CAALFM_C302270WA | 0.449 | 0.000572036 | yes | down |
| gene4714 | ARG3 | 0.481 | 0.000642741 | yes | down |
| gene6165 | CAALFM_CR09810WA | 0.373 | 0.000682013 | yes | down |
| gene1534 | ESC4 | 0.415 | 0.000705282 | yes | down |
| gene1519 | CAALFM_C201240CA | 0.436 | 0.000747293 | yes | down |
| gene2729 | CAALFM_C303020WA | 0.494 | 0.000808156 | yes | down |
| gene606 | CAALFM_C106340WA | 0.462 | 0.000857717 | yes | down |
| gene3939 | CAALFM_C500510WA | 0.475 | 0.000866305 | yes | down |
| gene222 | PGA5 | 0.46 | 0.000960386 | yes | down |
| gene2031 | CAALFM_C206530WA | 0.422 | 0.00102455 | yes | down |
| gene1774 | CAALFM_C203890WA | 0.496 | 0.001176606 | yes | down |
| gene687 | CAALFM_C107150WA | 0.483 | 0.001211077 | yes | down |
| gene2603 | CAALFM_C301740CA | 0.354 | 0.001252491 | yes | down |
| gene3660 | FAV1 | 0.452 | 0.001310631 | yes | down |
| gene1261 | CAALFM_C113100WA | 0.415 | 0.00146211 | yes | down |
| gene76 | CAALFM_C100820WA | 0.478 | 0.001686502 | yes | down |
| gene5867 | CAALFM_CR06630WA | 0.367 | 0.001836969 | yes | down |
| gene903 | LIP2 | 0.472 | 0.001840774 | yes | down |
| gene5051 | CAALFM_C702210WA | 0.355 | 0.001855646 | yes | down |
| gene4251 | CAALFM_C503880CA | 0.497 | 0.001958764 | yes | down |
| gene3391 | PHO89 | 0.405 | 0.002200956 | yes | down |
| gene3588 | CAALFM_C404040WA | 0.353 | 0.002255231 | yes | down |
| gene2553 | UTP20 | 0.486 | 0.002354984 | yes | down |
| gene569 | CAALFM_C105920WA | 0.5 | 0.002497593 | yes | down |
| gene4052 | CAALFM_C501710CA | 0.5 | 0.002810503 | yes | down |
| gene4054 | CAALFM_C501730WA | 0.461 | 0.002838777 | yes | down |
| gene3787 | MPH1 | 0.48 | 0.00288937 | yes | down |
| gene4482 | CTR1 | 0.417 | 0.00381871 | yes | down |
| gene4343 | CAALFM_C504840CA | 0.437 | 0.003885188 | yes | down |
| gene2367 | CAALFM_C210070WA | 0.468 | 0.003908898 | yes | down |
| gene3602 | CAALFM_C404190CA | 0.473 | 0.004875965 | yes | down |
| gene551 | CAALFM_C105740CA | 0.485 | 0.005760338 | yes | down |
| gene378 | CAALFM_C103960CA | 0.411 | 0.005849716 | yes | down |
| gene6199 | PGA12 | 0.494 | 0.006201155 | yes | down |
| gene2662 | CAALFM_C302330CA | 0.467 | 0.006773825 | yes | down |
| gene5581 | CAALFM_CR03580CA | 0.39 | 0.007374441 | yes | down |
| gene1489 | PGA19 | 0.474 | 0.008173955 | yes | down |
| gene1291 | OFI1 | 0.434 | 0.009357422 | yes | down |
| gene6168 | FGR46 | 0.426 | 0.009348268 | yes | down |
| gene4282 | MRV3 | 0.446 | 0.0121291 | yes | down |
| gene5417 | PTR2 | 0.447 | 0.012388415 | yes | down |
| gene1057 | CAALFM_C110980WA | 0.442 | 0.013086906 | yes | down |
| gene2526 | ATO1 | 0.427 | 0.013800788 | yes | down |
| gene1638 | ATO7 | 0.43 | 0.014045123 | yes | down |
| gene2226 | CAALFM_C208580WA | 0.451 | 0.014081126 | yes | down |
| gene5494 | CAALFM_CR02680WA | 0.438 | 0.015565957 | yes | down |
| gene3701 | CAALFM_C405230CA | 0.452 | 0.017348556 | yes | down |
| gene4306 |  | 0.469 | 0.017468769 | yes | down |
| gene978 | CAALFM_C110170WA | 0.42 | 0.017954308 | yes | down |
| gene3335 | CAALFM_C401340WA | 0.491 | 0.018835329 | yes | Down |
| gene5430 | HEM1 | 6.312 | 2.258E-137 | yes | up |
| gene2024 | RTA3 | 11.12 | 8.27837E-74 | yes | up |
| gene6101 | CAALFM_CR09100CA | 14.197 | 5.93765E-73 | yes | up |
| gene1701 | AMO1 | 8.261 | 1.64649E-72 | yes | up |
| gene4772 | SNQ2 | 10.586 | 3.8447E-71 | yes | up |
| gene2184 | CAALFM_C208100WA | 7.239 | 6.7054E-67 | yes | up |
| gene883 | CAALFM_C109210CA | 5.964 | 3.43262E-64 | yes | up |
| gene1128 | MRF1 | 7.124 | 5.19311E-62 | yes | up |
| gene4950 | CAALFM_C701130CA | 6.207 | 2.20571E-60 | yes | up |
| gene2397 | FGR6-4 | 5.675 | 2.69321E-60 | yes | up |
| gene395 | IFD6 | 10.239 | 1.02344E-59 | yes | up |
| gene6107 | SSU1 | 5.368 | 3.36678E-46 | yes | up |
| gene1131 | CAALFM_C111730WA | 2.847 | 8.37668E-40 | yes | up |
| gene382 | CAALFM_C104010CA | 3.919 | 2.2721E-37 | yes | up |
| gene3658 | MNN22 | 5.361 | 1.38879E-36 | yes | up |
| gene2280 | MET10 | 3.76 | 2.1357E-36 | yes | up |
| gene1996 | ECM17 | 3.419 | 2.08599E-36 | yes | up |
| gene874 | IPT1 | 2.91 | 7.05329E-36 | yes | up |
| gene2657 | GFA1 | 4.329 | 2.19877E-35 | yes | up |
| gene2939 | CDR1 | 2.809 | 9.32423E-34 | yes | up |
| gene446 | DUR1,2 | 4.932 | 5.55316E-33 | yes | up |
| gene2853 | ATF1 | 3.446 | 1.09119E-32 | yes | up |
| gene877 | AOX2 | 6.021 | 3.21523E-32 | yes | up |
| gene132 | CRL1 | 2.861 | 1.54389E-31 | yes | up |
| gene1683 | MET1 | 6.381 | 2.81929E-30 | yes | up |
| gene2772 | CAALFM_C303460CA | 6.468 | 5.83183E-30 | yes | up |
| gene1366 | IML2 | 3.214 | 2.53342E-27 | yes | up |
| gene3568 | HRQ2 | 4.546 | 6.01221E-27 | yes | up |
| gene5057 | CAALFM_C702280WA | 7.465 | 2.12904E-26 | yes | up |
| gene5055 | CAALFM_C702260WA | 7.577 | 2.31117E-26 | yes | up |
| gene2119 | RCL1 | 3.536 | 2.95983E-26 | yes | up |
| gene2308 | PMS1 | 5.65 | 5.8398E-26 | yes | up |
| gene2789 | ECM18 | 3.899 | 2.83529E-25 | yes | up |
| gene2740 | CAALFM_C303130CA | 3.899 | 2.83529E-25 | yes | up |
| gene2023 |  | 14.744 | 1.40136E-24 | yes | up |
| gene2360 | YOR1 | 3.625 | 1.62066E-24 | yes | up |
| gene72 | HGC1 | 6.008 | 1.69343E-24 | yes | up |
| gene996 | CAALFM_C110350CA | 2.526 | 6.71994E-24 | yes | up |
| gene1885 | CAALFM_C205040CA | 3.134 | 1.0705E-23 | yes | up |
| gene3944 | CAALFM_C500570WA | 3.441 | 2.28494E-23 | yes | up |
| gene2797 | GTT11 | 2.387 | 2.55961E-23 | yes | up |
| gene1262 | CHS3 | 3.107 | 3.50446E-23 | yes | up |
| gene4792 | CAALFM_C604100WA | 5.724 | 4.3047E-23 | yes | up |
| gene669 | CAALFM_C106970CA | 2.86 | 6.65854E-23 | yes | up |
| gene2182 | IFM3 | 3.067 | 1.80361E-22 | yes | up |
| gene2820 | AAP1 | 3.382 | 4.24809E-22 | yes | up |
| gene4477 | FAT1 | 2.315 | 4.46656E-22 | yes | up |
| gene4795 | ALS4 | 4.142 | 4.7372E-22 | yes | up |
| gene5263 | ALK2 | 3.278 | 4.88531E-22 | yes | up |
| gene2507 | CHS8 | 2.366 | 6.65286E-22 | yes | up |
| gene1133 | AVT4 | 3.321 | 6.60534E-22 | yes | up |
| gene4661 | WSC2 | 2.888 | 1.22378E-21 | yes | up |
| gene3780 | GDH3 | 3.606 | 1.25566E-21 | yes | up |
| gene3591 | PGA31 | 6.928 | 1.29639E-21 | yes | up |
| gene5217 | CAALFM_C704090CA | 3.082 | 1.49018E-21 | yes | up |
| gene4128 | UAP1 | 2.746 | 1.91608E-21 | yes | up |
| gene2861 | CAALFM_C304420WA | 2.293 | 3.75489E-21 | yes | up |
| gene2828 | CDR11 | 4.151 | 9.76381E-21 | yes | up |
| gene3275 | CAALFM_C400700CA | 2.694 | 1.0128E-20 | yes | up |
| gene4604 | CAALFM_C602100WA | 6.95 | 1.07046E-20 | yes | up |
| gene3718 | CAALFM_C405400CA | 3.271 | 1.72938E-20 | yes | up |
| gene4686 | CAALFM_C602940CA | 2.886 | 2.70448E-20 | yes | up |
| gene938 | CAALFM_C109770WA | 2.831 | 2.80964E-20 | yes | up |
| gene1978 | CAALFM_C205980CA | 2.199 | 4.95528E-20 | yes | up |
| gene5520 | CAALFM_CR02970CA | 2.235 | 1.76047E-19 | yes | up |
| gene6095 | CHS2 | 2.957 | 2.38569E-19 | yes | up |
| gene5798 | CAALFM_CR05860WA | 2.706 | 2.95683E-19 | yes | up |
| gene2576 | FOX3 | 3.558 | 6.08589E-19 | yes | up |
| gene554 | PRC3 | 3.428 | 8.56013E-19 | yes | up |
| gene1258 | CAALFM_C113060CA | 2.7 | 1.23386E-18 | yes | up |
| gene5916 | ALK8 | 3.832 | 2.56009E-18 | yes | up |
| gene568 | SIP5 | 2.58 | 5.54556E-18 | yes | up |
| gene937 | SHY1 | 2.666 | 1.18626E-17 | yes | up |
| gene1548 | CAALFM_C201540WA | 2.309 | 1.37753E-17 | yes | up |
| gene6046 | PGA13 | 4.246 | 1.68293E-17 | yes | up |
| gene5105 | PMT1 | 2.081 | 1.85459E-17 | yes | up |
| gene2334 | IPK2 | 2.471 | 2.43036E-17 | yes | up |
| gene2299 | TAZ1 | 3.712 | 2.95607E-17 | yes | up |
| gene4758 | ALS1 | 3.385 | 4.96026E-17 | yes | up |
| gene5254 | MKC1 | 3.029 | 8.648E-17 | yes | up |
| gene5483 | CAALFM_CR02570CA | 3.141 | 9.97818E-17 | yes | up |
| gene282 | GOR1 | 2.716 | 1.06212E-16 | yes | up |
| gene672 | MEF2 | 2.231 | 1.10195E-16 | yes | up |
| gene5056 | LDG3 | 5.527 | 1.19698E-16 | yes | up |
| gene6153 | PLB3 | 2.152 | 2.69221E-16 | yes | up |
| gene1474 | CAALFM_C200760CA | 3.747 | 3.05742E-15 | yes | up |
| gene57 | CAALFM_C100630WA | 2.511 | 7.57771E-15 | yes | up |
| gene114 | CAALFM_C101210WA | 2.336 | 8.59E-15 | yes | up |
| gene1888 | FRE9 | 2.999 | 9.44372E-15 | yes | up |
| gene3648 | CAALFM_C404670CA | 2.162 | 1.24516E-14 | yes | up |
| gene2746 | CAALFM_C303190CA | 2.507 | 1.63669E-14 | yes | up |
| gene3998 | CYC3 | 2.22 | 1.93961E-14 | yes | up |
| gene1032 | CAALFM_C110730WA | 2.379 | 1.95892E-14 | yes | up |
| gene5534 | CAALFM_CR03110WA | 2.422 | 2.84179E-14 | yes | up |
| gene782 | BUL1 | 2.543 | 3.12346E-14 | yes | up |
| gene1693 | PLC2 | 2.252 | 3.60457E-14 | yes | up |
| gene905 | CAALFM_C109440WA | 2.162 | 4.06675E-14 | yes | up |
| gene3291 | CAALFM_C400860CA | 2.592 | 5.57978E-14 | yes | up |
| gene5739 | GUT1 | 2.468 | 9.15202E-14 | yes | up |
| gene4741 | SAP4 | 3.637 | 9.50241E-14 | yes | up |
| gene4919 | CAALFM_C700770WA | 2.515 | 1.0833E-13 | yes | up |
| gene3614 | COX11 | 2.538 | 1.14884E-13 | yes | up |
| gene3036 | CYM1 | 2.251 | 1.51169E-13 | yes | up |
| gene3725 | HOM3 | 2.463 | 1.97999E-13 | yes | up |
| gene5315 | BMT3 | 2.497 | 2.8064E-13 | yes | up |
| gene104 | CCH1 | 2.071 | 4.59159E-13 | yes | up |
| gene4463 | SLY41 | 2.312 | 5.622E-13 | yes | up |
| gene5913 | FLC2 | 2.723 | 7.19129E-13 | yes | up |
| gene2568 | PGA44 | 3.424 | 7.16626E-13 | yes | up |
| gene2792 | IRS4 | 2.676 | 7.92625E-13 | yes | up |
| gene5859 | CAALFM_CR06550CA | 2.954 | 9.41236E-13 | yes | up |
| gene3401 | CAALFM_C402040WA | 2.115 | 1.00128E-12 | yes | up |
| gene4820 | ALS2 | 2.767 | 1.02856E-12 | yes | up |
| gene3390 | CAALFM_C401930CA | 2.497 | 1.04701E-12 | yes | up |
| gene1482 | CAALFM_C200840WA | 3.103 | 1.35078E-12 | yes | up |
| gene1979 | CAALFM_C205990CA | 2.585 | 1.69915E-12 | yes | up |
| gene3306 | DAG7 | 2.65 | 2.78015E-12 | yes | up |
| gene5922 | CAALFM_CR07190WA | 2.15 | 3.08252E-12 | yes | up |
| gene4740 | SAP1 | 2.396 | 3.18216E-12 | yes | up |
| gene4532 | CAALFM_C601340CA | 11.35 | 4.64853E-12 | yes | up |
| gene4823 | CAALFM_C604420WA | 2.539 | 1.0439E-11 | yes | up |
| gene2635 | CAALFM_C302060WA | 4.313 | 1.14766E-11 | yes | up |
| gene4440 | CAALFM_C600340CA | 2.057 | 2.97403E-11 | yes | up |
| gene5923 | CAALFM_CR07200WA | 2.241 | 4.14347E-11 | yes | up |
| gene413 | RPN4 | 2.761 | 4.20455E-11 | yes | up |
| gene2854 | CAALFM_C304350CA | 2.287 | 4.43262E-11 | yes | up |
| gene3287 | CAALFM_C400820WA | 2.512 | 4.55621E-11 | yes | up |
| gene629 | ANT1 | 2.265 | 4.67E-11 | yes | up |
| gene2708 | FAA21 | 2.557 | 4.70824E-11 | yes | up |
| gene2025 | RTA2 | 2.493 | 4.89026E-11 | yes | up |
| gene794 | OPY2 | 2.433 | 6.02307E-11 | yes | up |
| gene2605 | AFG3 | 2.032 | 6.43412E-11 | yes | up |
| gene4735 | CAALFM_C603440WA | 2.626 | 6.85735E-11 | yes | up |
| gene3624 | LYS4 | 2.702 | 7.46284E-11 | yes | up |
| gene3429 | SOD1 | 2.957 | 7.98312E-11 | yes | up |
| gene4830 | LIP4 | 2.076 | 8.27637E-11 | yes | up |
| gene3410 | CYT2 | 2.405 | 9.53296E-11 | yes | up |
| gene3524 | CAALFM_C403370CA | 2.673 | 1.2014E-10 | yes | up |
| gene1468 | CAALFM_C200700WA | 2.359 | 1.24794E-10 | yes | up |
| gene2132 | CAALFM_C207580WA | 2.418 | 1.36716E-10 | yes | up |
| gene5657 | PIM1 | 2.203 | 1.42679E-10 | yes | up |
| gene5445 | UGT51C1 | 2.314 | 1.85718E-10 | yes | up |
| gene6233 | YHB4 | 2.113 | 2.22702E-10 | yes | up |
| gene772 | CDR4 | 3.069 | 2.33721E-10 | yes | up |
| gene5638 | CAALFM_CR04150WA | 2.223 | 2.47765E-10 | yes | up |
| gene1866 | CAALFM_C204820WA | 2.106 | 2.84403E-10 | yes | up |
| gene2907 | CDR2 | 2.129 | 2.86785E-10 | yes | up |
| gene2661 | ILV2 | 2.165 | 3.11514E-10 | yes | up |
| gene5368 | CPA2 | 2.554 | 3.38385E-10 | yes | up |
| gene4884 | LEU2 | 2.464 | 3.56469E-10 | yes | up |
| gene3423 | CAALFM_C402260CA | 3.558 | 3.67997E-10 | yes | up |
| gene49 | CIS2 | 2.351 | 4.16712E-10 | yes | up |
| gene3766 | CAALFM_C405980CA | 2.061 | 4.87869E-10 | yes | up |
| gene4347 | PUT2 | 3.122 | 5.86744E-10 | yes | up |
| gene6189 | INO1 | 2.526 | 6.08996E-10 | yes | up |
| gene5850 | GST3 | 3.428 | 8.13982E-10 | yes | up |
| gene510 | CAALFM_C105330CA | 2.234 | 9.40194E-10 | yes | up |
| gene3025 | CAALFM_C306110CA | 2.049 | 9.50969E-10 | yes | up |
| gene383 | CSH1 | 2.126 | 9.73457E-10 | yes | up |
| gene4490 | ILV5 | 2.411 | 9.9679E-10 | yes | up |
| gene14 | LEU4 | 2.988 | 1.33507E-09 | yes | up |
| gene277 | MST1 | 2.057 | 2.17662E-09 | yes | up |
| gene4945 | CAALFM_C701040CA | 2.22 | 2.23939E-09 | yes | up |
| gene620 | CAALFM_C106480CA | 2.241 | 2.33078E-09 | yes | up |
| gene4028 | PRC2 | 2.207 | 2.63079E-09 | yes | up |
| gene247 | EXG2 | 2.227 | 2.90582E-09 | yes | up |
| gene1772 | GNA1 | 2.19 | 2.97464E-09 | yes | up |
| gene3783 | CAALFM_C406150CA | 7.574 | 3.13921E-09 | yes | up |
| gene4227 | BTS1 | 2.224 | 3.55225E-09 | yes | up |
| gene2660 | MEP1 | 2.283 | 5.35595E-09 | yes | up |
| gene5838 | CAALFM_CR06270WA | 3.317 | 6.89065E-09 | yes | up |
| gene4949 | CAALFM_C701100CA | 2.396 | 8.86665E-09 | yes | up |
| gene5774 | MTG2 | 2.035 | 1.07448E-08 | yes | up |
| gene4736 | NAM2 | 2.057 | 1.47433E-08 | yes | up |
| gene3414 | CAALFM_C402170CA | 2.855 | 2.96695E-08 | yes | up |
| gene2255 | PIR1 | 2.353 | 3.46255E-08 | yes | up |
| gene2183 | CAALFM_C208090WA | 2.873 | 3.95551E-08 | yes | up |
| gene1977 | TES1 | 2.352 | 4.25625E-08 | yes | up |
| gene1867 | CAALFM_C204830WA | 2.153 | 4.63602E-08 | yes | up |
| gene1969 | IDP1 | 2.309 | 5.09401E-08 | yes | up |
| gene891 | ARG5,6 | 2.087 | 5.35748E-08 | yes | up |
| gene4286 | MRV8 | 2.196 | 6.24877E-08 | yes | up |
| gene2342 | CAALFM_C209810CA | 2.057 | 6.32222E-08 | yes | up |
| gene977 | MIR1 | 2.011 | 6.73455E-08 | yes | up |
| gene1592 | CHT4 | 2.15 | 6.91147E-08 | yes | up |
| gene4746 | POX1 | 2.04 | 8.56761E-08 | yes | up |
| gene3286 | CAALFM_C400810CA | 3.303 | 1.02635E-07 | yes | up |
| gene3724 | FGR6-10 | 2.383 | 1.57804E-07 | yes | up |
| gene3398 | HSX11 | 2.179 | 1.78163E-07 | yes | up |
| gene902 | FTH1 | 2.344 | 1.83694E-07 | yes | up |
| gene5845 | CAALFM_CR06380CA | 2.372 | 2.05143E-07 | yes | up |
| gene1684 | CAALFM_C202950WA | 2.217 | 2.11276E-07 | yes | up |
| gene1747 | FMO2 | 2.159 | 2.21749E-07 | yes | up |
| gene839 | CAALFM_C108770WA | 2.309 | 2.44434E-07 | yes | up |
| gene2078 | CAALFM_C207020CA | 2.181 | 2.97798E-07 | yes | up |
| gene3583 | CAALFM_C403990CA | 2.293 | 6.18708E-07 | yes | up |
| gene5791 | ACO2 | 2.178 | 7.31333E-07 | yes | up |
| gene5000 | CAALFM_C701670WA | 2.015 | 7.40266E-07 | yes | up |
| gene5435 | CAALFM_CR02060WA | 2.018 | 8.13672E-07 | yes | up |
| gene431 | CAALFM_C104510WA | 2.214 | 9.6794E-07 | yes | up |
| gene1461 | CAALFM_C200620CA | 2.121 | 1.01539E-06 | yes | up |
| gene502 | CAALFM_C105250WA | 2.252 | 1.03772E-06 | yes | up |
| gene985 | CAALFM_C110240CA | 2.523 | 1.10706E-06 | yes | up |
| gene2837 | DAL8 | 2.022 | 1.27926E-06 | yes | up |
| gene5197 | CAALFM_C703880CA | 2.426 | 2.17776E-06 | yes | up |
| gene5876 | NMD3 | 2.017 | 3.24374E-06 | yes | up |
| gene1865 | HIS3 | 2.198 | 3.26683E-06 | yes | up |
| gene4990 | CAALFM_C701570CA | 3.261 | 4.37765E-06 | yes | up |
| gene3665 | AGP3 | 2.075 | 4.42163E-06 | yes | up |
| gene784 | CAT8 | 2.193 | 4.84383E-06 | yes | up |
| gene4747 | CAALFM_C603570WA | 3.122 | 6.66464E-06 | yes | up |
| gene319 | RLI1 | 2.006 | 9.52104E-06 | yes | up |
| gene3405 | CAALFM_C402080WA | 2.727 | 9.99633E-06 | yes | up |
| gene1170 | CAALFM_C112140WA | 2.109 | 1.1287E-05 | yes | up |
| gene1827 | IFA4 | 2.172 | 1.2877E-05 | yes | up |
| gene574 | CAALFM_C105970WA | 4.711 | 1.45265E-05 | yes | up |
| gene6062 | CAALFM_CR08670CA | 2.104 | 1.52963E-05 | yes | up |
| gene1893 | CAALFM_C205130WA | 2.13 | 1.58845E-05 | yes | up |
| gene1348 | CAALFM_C114020WA | 3.422 | 2.05814E-05 | yes | up |
| gene1831 | LYS22 | 2.142 | 2.08152E-05 | yes | up |
| gene1975 | TES15 | 2.221 | 2.72658E-05 | yes | up |
| gene1653 | CAALFM_C202630WA | 2.174 | 3.06805E-05 | yes | up |
| gene213 | CAALFM_C102270CA | 2.227 | 3.24399E-05 | yes | up |
| gene5424 | BIO2 | 2.3 | 3.87677E-05 | yes | up |
| gene5981 | YHB5 | 2.237 | 4.35709E-05 | yes | up |
| gene1259 | FAD3 | 2.148 | 8.12798E-05 | yes | up |
| gene5517 | CAALFM_CR02940CA | 2.184 | 0.000103682 | yes | up |
| gene4495 | CAALFM_C600920WA | 2.014 | 0.000112443 | yes | up |
| gene3419 | ZCF25 | 2.193 | 0.000171825 | yes | up |
| gene1597 | FMO1 | 2.034 | 0.000214031 | yes | up |
| gene3971 | CAALFM_C500850CA | 2.044 | 0.000246565 | yes | up |
| gene5648 | CAALFM_CR04250WA | 2.31 | 0.00030798 | yes | up |
| gene566 | CAALFM_C105890WA | 3.619 | 0.000368232 | yes | up |
| gene2501 | NGT1 | 2.172 | 0.000385405 | yes | up |
| gene5093 |  | 2.459 | 0.000411225 | yes | up |
| gene1613 | CAALFM_C202220CA | 2.515 | 0.000456315 | yes | up |
| gene5098 | PRX1 | 2.211 | 0.000482515 | yes | up |
| gene5054 | CAALFM_C702250WA | 2.219 | 0.000504637 | yes | up |
| gene615 | CAALFM_C106430CA | 2.929 | 0.000978698 | yes | up |
| gene742 |  | 2.119 | 0.000986389 | yes | up |
| gene1502 | CAALFM_C201060CA | 2.671 | 0.001200785 | yes | up |
| gene3035 | OAC1 | 3.098 | 0.001203781 | yes | up |
| gene718 | CAALFM_C107480CA | 2.226 | 0.002162566 | yes | up |
| gene3729 | CAALFM_C405580CA | 2.927 | 0.003431163 | yes | up |
| gene3071 | JEN1 | 2.66 | 0.003802745 | yes | up |
| gene1333 | MET3 | 2.689 | 0.003903374 | yes | up |
| gene1829 | CAALFM_C204440WA | 2.782 | 0.005314041 | yes | up |
| gene1130 | CAALFM_C111720WA | 2.673 | 0.005416901 | yes | up |
| gene5216 | CAALFM_C704080CA | 2.181 | 0.011343421 | yes | up |
| gene609 | PBR1 | 2.055 | 0.018194043 | yes | up |
